# Lactate released by inflammatory bone marrow neutrophils induces their mobilization via endothelial GPR81 signaling

**DOI:** 10.1038/s41467-020-17402-2

**Published:** 2020-07-15

**Authors:** Eman Khatib-Massalha, Suditi Bhattacharya, Hassan Massalha, Adi Biram, Karin Golan, Orit Kollet, Anju Kumari, Francesca Avemaria, Ekaterina Petrovich-Kopitman, Shiri Gur-Cohen, Tomer Itkin, Isabell Brandenburger, Asaf Spiegel, Ziv Shulman, Zachary Gerhart-Hines, Shalev Itzkovitz, Matthias Gunzer, Stefan Offermanns, Ronen Alon, Amiram Ariel, Tsvee Lapidot

**Affiliations:** 1 Department of Immunology, Weizmann Institute of Science, Rehovot, Israel; 2 Department of Molecular Cell Biology, Weizmann Institute of Science, Rehovot, Israel; 3 Life science Core facilities, Weizmann Institute of Science, Rehovot, Israel; 4 Department of Pharmacology, Max-Planck-Institute for Heart and Lung Research, Bad Nauheim, Germany; 5 Novo Nordisk Foundation Center for Basic Metabolic Research, University of Copenhagen, Copenhagen, Denmark; 6 Institute for Experimental Immunology and Imaging, University Hospital, University Duisburg-Essen, Essen, Germany; 7 Department of Human Biology, University of Haifa, Haifa, Israel

**Keywords:** Bacterial infection, Acute inflammation, Neutrophils, Metabolism

## Abstract

Neutrophils provide first line of host defense against bacterial infections utilizing glycolysis for their effector functions. How glycolysis and its major byproduct lactate are triggered in bone marrow (BM) neutrophils and their contribution to neutrophil mobilization in acute inflammation is not clear. Here we report that bacterial lipopolysaccharides (LPS) or *Salmonella* Typhimurium triggers lactate release by increasing glycolysis, NADPH-oxidase-mediated reactive oxygen species and HIF-1α levels in BM neutrophils. Increased release of BM lactate preferentially promotes neutrophil mobilization by reducing endothelial VE-Cadherin expression, increasing BM vascular permeability via endothelial lactate-receptor GPR81 signaling. GPR81^−/−^ mice mobilize reduced levels of neutrophils in response to LPS, unless rescued by VE-Cadherin disrupting antibodies. Lactate administration also induces release of the BM neutrophil mobilizers G-CSF, CXCL1 and CXCL2, indicating that this metabolite drives neutrophil mobilization via multiple pathways. Our study reveals a metabolic crosstalk between lactate-producing neutrophils and BM endothelium, which controls neutrophil mobilization under bacterial infection.

## Abstract (web summary)

Lactate is a by-product of glycolysis that can function via its G protein receptor GPR81. Here the authors show that LPS or *Salmonella* infection enhances glycolytic metabolism in bone marrow neutrophils, resulting in lactate production, which increases endothelial barrier permeability and mobilization of these neutrophils by targeting endothelial GPR81.

## Introduction

Innate immune neutrophils are the first cells to be mobilized and recruited from the bone marrow (BM), against bacterial infections^1^. During steady-state homeostasis, most murine neutrophils reside in the BM, and only a small fraction migrate to the circulation^2,3^. However, during inflammation, BM neutrophils are rapidly mobilized to the blood and recruited to peripheral organs in order to eradicate the invading pathogens^4,5^. Activation of pro-inflammatory signaling pathways involves stimulation of metabolic pathways in immune cells as a part of host defense responses^6^. These include increased oxygen consumption during myeloid induced phagocytosis, due to activation of NADPH oxidase (NOX) in order to generate oxygen free radicals^7^. Furthermore, studies mimicking bacterial infection also revealed increased glucose uptake^8^ and a shift to glycolysis rather than oxidative phosphorylation, which is characteristic of pro-inflammatory macrophages^9^.

While there are multiple studies concerning immune-metabolism of macrophages, less is known about neutrophil metabolic control during the onset of acute inflammation. Neutrophils are highly glycolytic with limited mitochondrial respiration^10,11^. Lactate, a major byproduct of glycolysis, was found to be released by human neutrophils^12^ and increased lactate levels are used as a marker for detecting sepsis in patients^13,14^. However, mechanistic reasoning for lactate production by BM neutrophils and its potential role during the early phase of acute inflammation remain elusive.

Here, we report that exposure to LPS or live *Salmonella* activates (within 4 h) BM neutrophils to produce and release lactate in both NOX- and hypoxia-inducible factor-1α (HIF-1α)- dependent manners. The metabolite lactate preferentially mobilizes neutrophils by increasing BM vascular permeability upon activation of the lactate-receptor GPR81 expressed by BM endothelial cells. In addition, lactate also induces the release of the neutrophil attracting chemokines CXCL1 and CXCL2, and of the neutrophil mobilizing-cytokine granulocyte colony stimulating factor (G-CSF), which also involves GPR81-independent mechanisms. Consequently, lactate administration increases the defective LPS-induced mobilization of activated neutrophils in NOX-mutated mice, further demonstrating the critical roles of this metabolite in neutrophil mobilization during the early phase of bacterial infection.

## Results

### LPS increases lactate production by BM neutrophils

**Fig. 1:**
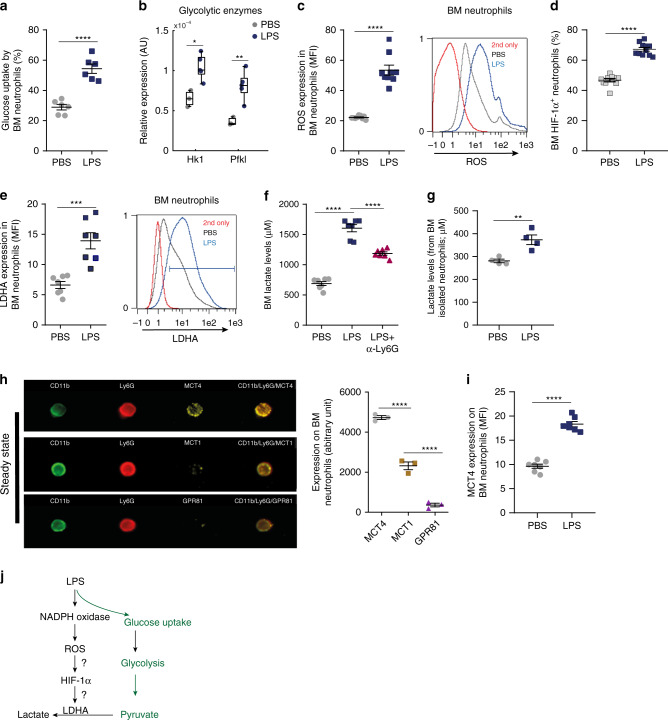
LPS increases glycolysis as well as lactate production by BM neutrophils. **a** Flow cytometry quantitative analysis of 2-NBDG-glucose uptake by BM neutrophils (CD11b^high^Ly6G^high^ cells; *n* = 6) 4 h following i.p. administration of LPS in vivo in wild-type (WT) mice. **b** Gene expression of glycolytic enzymes in sorted BM neutrophils from WT mice following LPS treatment (*n* = 3, PBS; *n* = 5, LPS). On each box, the bottom, middle, and the top edges indicate the 25th, 50th, and 75th percentiles, respectively. The whiskers extend to the most extreme data points. **c** Quantitative analysis and representative histogram plot showing mean fluorescent intensity (MFI) of ROS production in BM neutrophils following LPS administration (*n* = 9). **d** Percentage of HIF-1α^+^ neutrophils in the BM following LPS administration (*n* = 11). **e** Quantitative analysis and representative histogram plot of LDHA expression in BM neutrophils following LPS treatment (*n* = 7). ***p(0.0003). **f** BM lactate levels in WT mice treated with PBS, LPS, or LPS followed by α-Ly6G Ab (*n* = 7). **g** Lactate levels released from isolated BM neutrophils treated ex vivo with PBS or LPS (120 ng/ml; *n* = 4 mice).**p(0.0063). **h** MCT4, MCT1, and GPR81 (yellow) distribution on BM CD11b^+^ (green)/Ly6G^+^ (red) neutrophils visualized and quantified by ImageStream analysis. Images are from one representative experiment out of three. Scale bar indicates 7 μm. **i** Quantitative analysis of MCT4 expression on BM neutrophils 4 h following LPS administration (*n* = 7). **j** A scheme of the proposed mode of action of LPS in lactate production by BM neutrophils. Data are represented as mean ± SEM from 3 to 4 independent experiments. *p < 0.05; **p < 0.01; ***p < 0.001; ****p < 0.0001, Student’s two-tailed unpaired *t* test (**a**, **c**–**e**, **g**, **i**), one-way ANOVA with Tukey’s post hoc test (**f**, **h**) or two-way ANOVA with Tukey’s post hoc test (**b**). See also Supplementary Fig. 1.

Neutrophils are predominantly glycolytic cells that produce reactive oxygen species (ROS) through the cytosolic enzyme NOX. This process is essential for microbial eradication and regulation of inflammation^15,16^. To better understand the metabolic consequences of BM neutrophil activation during the onset of acute inflammation, we treated wild-type (WT) mice with a low dose of LPS to mimic acute gram-negative bacterial inflammation. Our findings indicate that 4 h after LPS administration activated BM neutrophils (CD11b^+^/Ly6G^high^ cells; Supplementary Fig. 1a) displayed increased glucose uptake (Fig. 1a), upregulated gene expression encoding the rate limiting glycolytic enzymes (hexokinase 1 (HK1) and phosphofructokinase 1 (PFKL); Fig. 1b) and downregulated levels of the TCA cycle genes (Supplementary Fig. 1b). Collectively, our findings suggest that BM neutrophils activate their glycolysis with very low rates of TCA cycle and oxidative phosphorylation during the onset of acute inflammation.

Next, we documented high production of ROS in BM neutrophils following LPS administration (Fig. 1c). Since ROS was shown to activate HIF-1α in macrophages^17^, we tested the impact of LPS on HIF-1α levels in BM neutrophils and found higher percentages of HIF-1α^+^ neutrophils in the BM induced by LPS exposure (Fig. 1d). Moreover, we found that BM neutrophils express elevated levels of lactate dehydrogenase A (LDHA), a key glycolytic enzyme involved in the conversion of pyruvate to lactate, following systemic exposure to LPS (Fig. 1e). Notably, we found that selective depletion of neutrophils by neutralizing Ly6G antibodies resulted in lower levels of BM lactate (a functional output of LDHA activity) in mice injected with LPS (Fig. 1f, Supplementary Fig. 1c). These data were supported by the observation that BM isolated neutrophils directly released high amounts of lactate following in vitro LPS stimulation (Fig. 1g, Supplementary Fig. 1d). Taken together, our results demonstrate that LPS can directly induce glycolysis and oxidative bursts in BM neutrophils which lead to the production and release of lactate by these leukocytes during the early phase of acute inflammation. However, we cannot rule out that LPS administration can also indirectly activate BM neutrophils and drive their mobilization via its effects on other BM cell subsets.

Lactate cellular levels are tightly balanced by monocarboxylase transporters (MCTs)^12,18^. MCT1 mediates lactate influx, while MCT4 is expressed only in glycolytic cells and mediates lactate efflux^19,20^. In addition, lactate can bind and signal through its G-protein coupled receptor GPR81 (also named hydroxycarboxylic acid receptor 1 (HCAR1)) and activates different signaling pathways^21,22^. We found that in steady state, MCT4 is highly expressed by BM neutrophils, while low expression of MCT1 and GPR81 was observed on the neutrophil surface (Fig. 1h, Supplementary Fig. 1e, f). Among BM myeloid cells, we found that MCT4 is preferentially expressed by neutrophils (LysM^high^/Ly6G^high^) and to a lower extent by monocytes (LysM^int^/Ly6G^neg^) (Supplementary Fig. 1g). Moreover, LPS administration further upregulated MCT4 expression on BM neutrophils (Fig. 1i). Interestingly, we found that LPS-induced glucose uptake (Supplementary Fig. 1h) and MCT4 expression (Supplementary Fig. 1i) also on PB neutrophils as well and not only on BM neutrophils. In addition, LPS treatment increased the levels of lactate (Supplementary Fig. 1j) and reduced the glucose levels in the blood without changing the blood pH (Table 1), suggesting that LPS-mobilized neutrophils are still glycolytic and most probably release lactate upon reaching vascular targets within inflamed peripheral organs to further promote their recruitment. Taken together, our results suggest that during the acute phase of LPS-induced inflammation, BM neutrophils uptake high amounts of glucose and convert it to lactate via glycolysis in the cytosol. Consequently, ROS and HIF-1α levels are increased to further activate the glycolytic pathway to produce and to release high levels of lactate via MCT4 (Fig. 1j).

**Table 1:** Effects of lactate or LPS treatments on blood pH and glucose levels in WT mice.

Mouse number	Gender/age	Treatment	Blood pH	Blood glucose (mg/dL)
#1	Male/8 weeks	PBS	6.739	337
#2	Male/8 weeks	PBS	6.753	300
#3	Male/8.5 weeks	PBS	6.778	249
#4	Male/8.5 weeks	PBS	6.829	251
Mean-PBS	NA	PBS	6.774	284.25
#4	Male/8 weeks	Lactate	6.854	225
#5	Male/8 weeks	Lactate	6.814	211
#6	Male/8 weeks	Lactate	6.863	177
#7	Male/8 weeks	Lactate	6.912	192
Mean-lactate	NA	Lactate	6.860^n.s.^	201.25*
#8	Male/8 weeks	LPS	6.962	131
#9	Male/8 weeks	LPS	6.976	127
#10	Male/8.5 weeks	LPS	6.743	183
#11	Male/8.5 weeks	LPS	6.767	172
Mean-LPS	NA	LPS	6.862 ^n.s.^	153.25***

Blood pH: n.s. for PBS compared with lactate or LPS. Blood glucose: ^*^p(0.0126) for PBS compared with lactate. ^***^p(0.0007) for PBS compared with LPS. One-way ANOVA with Tukey’s post hoc test.

### Lactate preferentially induces BM neutrophil mobilization

**Fig. 2:**
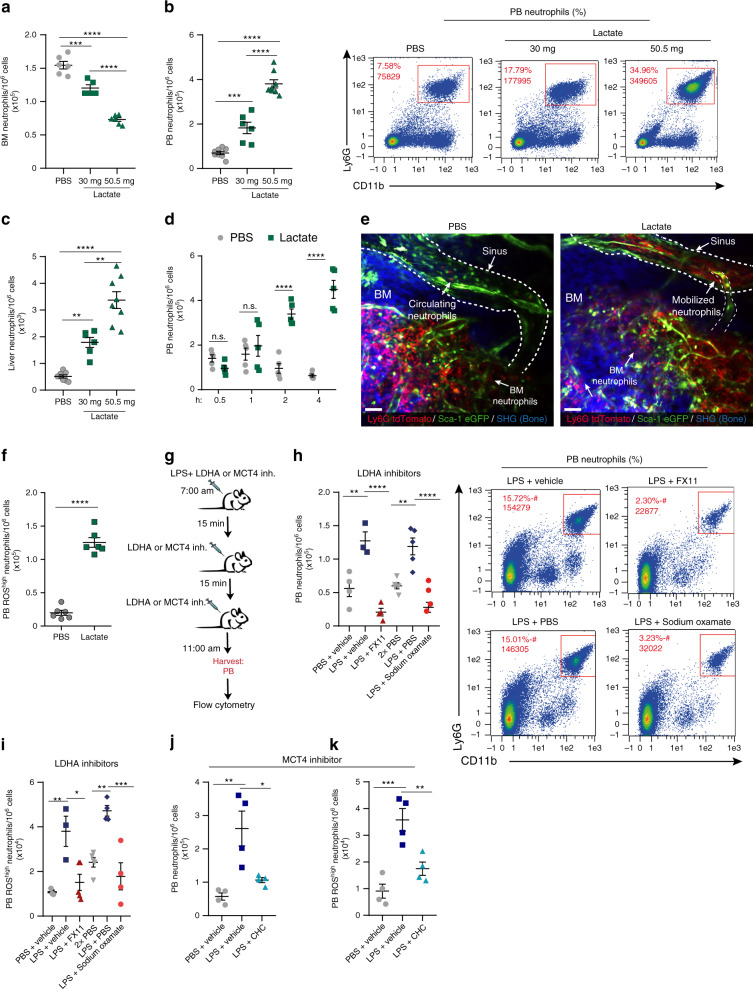
Lactate induces rapid BM neutrophil mobilization and recruitment. **a** BM neutrophil frequency (numbers per 10^6^ acquired cells by flow cytometry) 4 h following i.p. lactate treatment in vivo (*n* = 6, PBS or 50.5 mg lactate; *n* = 5, 30 mg lactate).***p (PBS vs. 30 mg lactate) = 0.004. **b**, **c** Quantitative analysis and representative flow cytometry density plots for **b** PB neutrophils and **c** liver neutrophils frequency (*n* = 8, PBS or 50.5 mg lactate; *n* = 6, 30 mg lactate) following lactate injection.***p(PBS vs. 30 mg lactate in the PB) = 0.0008; **p(PBS vs. 30 mg lactate in the liver) = 0.0023; ***p(30 mg lactate vs. 50 mg lactate in the liver) = 0.0003. **d** Neutrophil frequency in PB at different time points following 50.5 mg lactate treatment (*n* = 5). **e** Representative TPLSM 3D images 4 h post PBS (left panel) or lactate (right panel) treatment, in calvarial bone of chimeric mice reconstituted with Ly6G tdTomato neutrophils (red). Sca-1 eGFP marked the endothelial cells (green) and second harmonic generation marked the bone (SHG; blue). White dashed lines indicate superior sagittal sinus (calvaria central sinus), arrows indicate BM, circulating or mobilized neutrophils. Representative images out of three independent experiments are shown. Scale bar, 50 μm. **f** Quantitative analysis for PB ROS^high^ neutrophils frequency following lactate administration (*n* = 6). **g** A schematic illustration of the protocol for LPS with LDHA or MCT4 inhibitors administration. **h** Frequency of PB neutrophils following LDHA inhibitors (*n* = 4, PBS + vehicle; *n* = 3, LPS + vehicle; *n* = 4, LPS with FX11; *n* = 5, two injections of PBS or LPS + PBS; *n* = 4, LPS with sodium oxamate). **i** Frequency of PB ROS^high^ neutrophils following LDHA inhibitors (*n* = 3, PBS + vehicle or LPS + vehicle; *n* = 5, LPS with FX11; or two injections of PBS; *n* = 4, LPS + PBS or LPS with sodium oxamate). **j**, **k** Frequency of  **j** PB neutrophils or **k** PB ROS^high^ neutrophils following MCT4 inhibitor (*n* = 4). **j** **p(PBS + vehicle vs. LPS + vehicle) = 0.0032; *p(LPS + vehicle vs. LPS + CHC) = 0.0163; **k** ***p(PBS + vehicle vs. LPS + vehicle) = 0.0006; **p(LPS + vehicle vs. LPS + CHC) = 0.0073. Data are represented as mean ± SEM from 3 to 5 independent experiments. *p < 0.05; **p < 0.01; ***p < 0.001; ****p < 0.0001, one-way ANOVA with Tukey’s post hoc test (**a**–**c**, **h**–**k**), two-way ANOVA with Bonferroni post hoc test (**d**) or Student’s two-tailed unpaired *t* test (**f**). See also Supplementary Fig. 2.

Since our results indicate that BM neutrophils produce lactate during the onset of acute LPS-induced inflammation, we next investigated whether exogenous administration of this metabolite affects neutrophil mobilization from the BM, a hallmark of bacterial infection. Strikingly, injection of sodium lactate to WT mice (an upper physiological concentration range, Supplementary Fig. 2a, b) led to reduction in BM neutrophil levels within 4 h (Fig. 2a, Supplementary Fig. 2c), accompanied by robust neutrophil mobilization to the peripheral blood (PB; Fig. 2b, Supplementary Fig. 2d) and recruitment to the liver in a dose-dependent manner (Fig. 2c), without changing the blood pH (Table 1).

Notably, rapid neutrophil mobilization to the blood was detected as early as 2 h post lactate injection (Fig. 2d). We applied intravital two-photon laser-scanning microscopy in order to track live neutrophil mobilization over time. We generated chimeric mice in which hematopoietic cells from the BM of Catchup (Ly6G tdTomato^23^) mice were transplanted into Sca-1 eGFP recipient mice (Supplementary Fig. 2e), and tracked live neutrophils in the calvarial bone (Supplementary Fig. 2f). Consistent with our flow cytometry-based results, we observed high numbers of neutrophils (Ly6G positive tdTomato cells) within the superior sagittal sinus (sinusoid endothelial Sca-1^low^ cells^24^) of the calvarial BM ~3 or 4 h post lactate administration as indicated (Supplementary Fig. 2g, h, Supplementary Movies 1 and 2, Fig. 2e, Supplementary Fig. 2i).

Upon neutrophil activation, ROS are produced by NOX via a robust “oxidative burst”^25,26^. To assess if neutrophils acquire an activated phenotype in response to lactate, we measured ROS production and found increased levels of ROS^high^ activated neutrophils in the circulation 4 h after lactate administration (Fig. 2f). Next, we found that the number of white blood cells (WBCs) was reduced in the BM following lactate injection (Supplementary Fig. 3a). In addition, despite the increase in PB neutrophils, we detected reduction in the number of PB WBCs (Supplementary Fig. 3a), suggesting a differential cell-specific response. We therefore determined the effect of lactate on other BM immune cells. Our results reveal that despite the expression of lactate transporters MCT4 and MCT1 and lactate receptor GPR81 on other BM immune cells (Supplementary Fig. 3b–d; respectively), lactate administration acted preferentially on neutrophils (LysM^high^/Ly6G^high^ cells), as we found no significant increase in the levels of PB monocytes or lymphocytes 4 h post lactate injection (Supplementary Fig. 3e, f; respectively). These results suggest that in addition to the higher levels of lactate transporters, other pro-inflammatory factors are involved in the rapid neutrophil mobilization 4 h following lactate administration. Nevertheless, mobilization of monocytes to the circulation was observed later, 8 h post lactate injection (Supplementary Fig. 3g).

Finally, we examined the involvement of lactate production and release in neutrophil mobilization during acute inflammation. LPS administration increased the levels of PB neutrophils, in particular of activated ROS^high^ neutrophils (Fig. 2h–k). Pharmacological inhibition (Fig. 2g) of LDHA by specific inhibitors (sodium oxamate or FX11) or inhibition of MCT4 (lactate efflux) by α-cyano-4-hydroxycinnamic acid (CHC) markedly reduced the levels of either neutrophils or ROS^high^ neutrophils in the blood (Fig. 2h–k, respectively, see Methods for gating neutrophils in these specific experiments). Taken together, our results suggest that lactate preferentially induces rapid neutrophil mobilization to the peripheral blood and in particular of activated ROS^high^ neutrophils, followed by other myeloid cells, such as monocytes later on.

### Lactate production by neutrophils requires NOX/ROS signaling

**Fig. 3:**
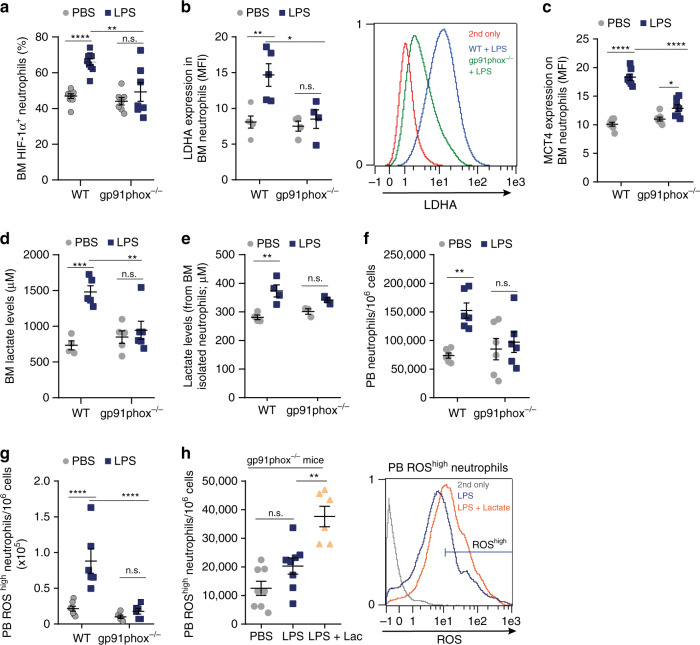
Lactate production by neutrophils requires NOX/ROS signaling. **a** Percentage of BM HIF-1α^+^ neutrophils in WT (*n* = 9) and gp91phox^−/−^ (*n* = 7) mice treated with either PBS or LPS. **p(WT + LPS vs. gp91phox^−/−^+LPS) = 0.0012. **b** Quantitative analysis and representative histogram plot showing LDHA expression in BM neutrophils from WT (*n* = 5) and gp91phox^−/−^ (*n* = 4) mice. **p(WT + PBS vs. WT + LPS) = 0.0054; *p(WT + LPS vs. gp91phox^−/−^+LPS) = 0.0131. **c** Quantitative analysis of MCT4 expression on BM neutrophils from WT (*n* = 7) and gp91phox^−/−^ (*n* = 7) mice treated with either PBS or LPS. *p(gp91phox^−/−^+PBS vs. gp91phox^−/−^+LPS) = 0.0424. **d** BM lactate levels in WT (*n* = 4, PBS; *n* = 5, LPS) and gp91phox^−/−^ (*n* = 5, PBS; *n* = 6, LPS) mice treated with LPS. **e** BM lactate levels released from WT or gp91phox^−/−^ isolated neutrophils treated ex vivo with PBS or LPS (120 ng/ml; *n* = 4, WT; *n* = 3, gp91phox^−/−^). **f**, **g** Frequency of PB neutrophils from WT and gp91phox^−/−^ (**f**, *n* = 6; **p(WT + PBS vs. WT + LPS) = 0.0058) and ROS^high^ neutrophils (**g**, *n* = 7, WT + PBS; *n* = 6, WT + LPS; *n* = 6, gp91phox^−/−^ mice) following LPS injection. **h** Quantitative analysis and representative histogram plot of PB ROS^high^ neutrophils of gp91phox^−/−^ mice treated with PBS, LPS, or LPS together with 50.5 mg lactate (*n* = 8, PBS or LPS; *n* = 6, LPS + lactate).**p(LPS vs. LPS + lactate) = 0.0018. Data are represented as mean ± SEM from 3 to 4 independent experiments. *p < 0.05; **p < 0.01; ***p < 0.001; ****p < 0.0001, one-way ANOVA with Tukey’s post hoc test (**h**) or two-way ANOVA with Tukey’s post hoc test (**a**–**g**). See also Supplementary Fig. 4.

The NOX/ROS axis in inflamed and activated neutrophils plays a key role in phagocytic defense against microbial pathogens. NOX also triggers ROS-mediated HIF-1α expression in macrophages^17^ and activates HIF-1α in prostate cancer^27^. Thus, we investigated whether the NOX/ROS axis is also involved in LPS-induced metabolic activation of BM neutrophils by treating gp91phox^−/−^ (NOX2 mutated) mice with LPS. We found a reduction in the levels of BM HIF-1α^+^ neutrophils compared to their WT counterparts (Fig. 3a). Interestingly, NOX/ROS deficiency in gp91phox^−/−^ mice impaired the molecular machinery imperative for lactate production and release by inflammatory BM neutrophils (indicated by the expression of LDHA and MCT4, and by BM lactate levels in vivo and in vitro) (Fig. 3b–e, respectively). These results suggest that the NOX/ROS axis is essential for HIF-1α activity and lactate production by BM neutrophils during acute LPS-induced inflammation.

To determine whether NOX expression in neutrophils or in BM stromal cells is essential for lactate production, we established chimeric mice in which hematopoietic cells from the BM of WT mice were transplanted into gp91phox^−/−^ mice and vice versa (Supplementary Fig. 4a). As expected, we found that following LPS administration the NOX/ROS signaling in neutrophils and not in the stromal compartment is responsible for HIF-1α elevation (Supplementary Fig. 4b) as well as lactate production and release (Supplementary Fig. 4c, d). In line with these findings, we found that the NOX mutation impairs PB neutrophil activation predominantly in chimeric mice with NOX mutated in the hematopoietic compartment (Supplementary Fig. 4e).

Consequently, LPS treatment failed to mobilize neutrophils in general and activated ROS^high^ neutrophils in particular to the blood in gp91phox^−/−^ mice compared to their WT counterparts (Fig. 3f, g). Importantly, lactate injection to bypass endogenous production and release of lactate by neutrophils, increased the levels of PB ROS^high^ neutrophils in gp91phox^−/−^ mice following LPS treatment (Fig. 3h). Taken together, our data reveal that NOX/ROS activities are upstream of lactate production in BM neutrophils since abnormal low metabolic rates were found in gp91phox^−/−^ neutrophils during the onset of the acute inflammation.

### HIF-1α mediates lactate release and neutrophil mobilization

Activation of HIF-1α is essential for myeloid cell activation, infiltration to inflamed tissues and for the regulation of the glycolytic capacity in these cells^28,29^. HIF-1α regulates macrophage metabolism by enhancing the expression of glycolytic enzymes^30–32^. However, the involvement of HIF-1α in neutrophil-derived lactate production and release during the early phase of acute LPS-triggered inflammation has not been demonstrated.

**Fig. 4:**
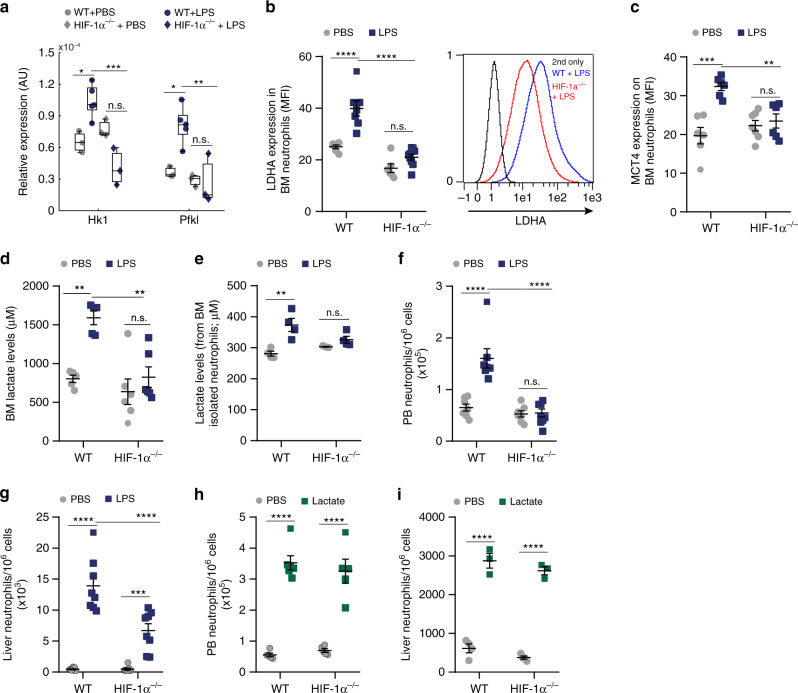
HIF-1α mediates lactate release and inflammatory neutrophil mobilization. **a** Gene expression of glycolytic enzymes in sorted BM neutrophils following LPS treatment (WT; *n* = 3, PBS; *n* = 5, LPS; HIF-1α^−/−^, *n* = 3). On each box, the bottom, middle and the top edges indicate the 25th, 50th, and 75th percentiles, respectively. The whiskers extend to the most extreme data points. **b** Quantitative analysis and representative flow cytometry histogram plot of LDHA expression in BM neutrophils in WT (*n* = 6, PBS; *n* = 7, LPS) vs. HIF-1α^−/−^ (*n* = 6, PBS; n = 9, LPS) mice. **c** Quantitative analysis of MCT4 expression on BM neutrophils following either PBS or LPS treatment in WT (*n* = 6) vs. HIF-1α^−/−^ (*n* = 7, PBS; *n* = 6, LPS) mice.***p(WT + PBS vs. WT + LPS) = 0.0001; **p(WT + LPS vs. HIF-1α^−/−^+LPS) = 0.0053. **d** BM lactate levels in WT (*n* = 5) vs. HIF-1α^−/−^ (*n* = 6) mice. **p(WT + PBS vs. WT + LPS) = 0.0023; **p(WT + LPS vs. HIF-1α^−/−^+LPS) = 0.0020. **e** BM Lactate levels released from WT or HIF-1α^−/−^ isolated neutrophils treated in vitro with PBS or LPS (120 ng/ml; *n* = 4 each group) **p(WT + PBS vs. WT + LPS) = 0.0027. **f** PB neutrophils frequency in WT (*n* = 7) and HIF-1α^−/−^ (*n* = 7, PBS; *n* = 8, LPS) mice. **g** Liver neutrophil frequency in WT (*n* = 8) and HIF-1α^−/−^ (*n* = 8) mice following LPS treatment. **h** PB neutrophil frequency in WT (*n* = 5, PBS; *n* = 6, lactate) and HIF-1α^−/−^ (*n* = 5) mice following 50.5 mg lactate treatment. **i** Liver neutrophil frequency in WT vs. HIF-1α^−/−^ (*n* = 4, PBS; *n* = 3, lactate) following lactate administration. Data are represented as mean ± SEM from 3 to 4 independent experiments. *p < 0.05; **p < 0.01; ***p < 0.001; ****p < 0.0001, two-way ANOVA with Bonferroni post hoc test (**a**) or two-way ANOVA with Tukey’s post hoc test (**b**–**i**). See also Supplementary Fig. 5.

To better understand the function of HIF-1α in lactate production by activated BM neutrophils, we generated mice with a myeloid-selective HIF-1α deficiency (deletion of exon 2 that disrupts the HIF-1α coding sequence; Supplementary Fig. 5a) and compared the expression of LDHA in BM neutrophils of WT, HIF-1α^−/+^ (heterozygous) and HIF-1α^−/−^ (homozygous) mice treated with LPS. Our results demonstrate that HIF-1α^−/−^ neutrophils displayed lower expression of the glycolytic enzymes (*HK1* and *PFKL*) compared to WT neutrophils following LPS treatment (Fig. 4a), suggesting that HIF-1α regulates the expression of rate limiting glycolytic enzymes during inflammation.

In addition, we also found a significant increased LDHA expression following LPS treatment in WT but not in HIF-1α^−/+^ or HIF-1α^−/−^ mice (Supplementary Fig. 5b, Fig. 4b, respectively). Another HIF-1α target is the lactate transporter MCT4^33^. In our myeloid-specific HIF-1α^−/+^ or HIF-1α^−/−^ mice, LPS treatment up-regulated within 4 h MCT4 expression on BM neutrophils in a HIF-1α dependent manner (Supplementary Fig. 5c and Fig. 4c). Moreover, BM HIF-1α mutated neutrophils failed to release lactate (Supplementary Fig. 5d, Fig. 4d, e) in contrast to WT BM neutrophils. Taken together, these results reveal that optimal neutrophil HIF-1α activity is essential for both high glycolysis activity and lactate production and release by activated BM neutrophils during the onset of acute LPS-triggered inflammation.

Next, we determined the role of HIF-1α in LPS-induced neutrophil mobilization to the circulation. We found lower levels of neutrophils in both the PB and in the liver of HIF-1α mutated mice treated with LPS (Supplementary Fig. 5e, f, Fig. 4f, g) in comparison with their WT counterparts. Notably, lactate injection to HIF-1α mutated mice increased neutrophil levels in both PB and liver, similar to WT mice (Supplementary Fig. 5g, h, Fig. 4h, i), thus placing HIF-1α upstream of lactate production.

Altogether, these findings indicate that HIF-1α directs metabolic programs in BM neutrophils, including glycolysis as well as lactate production and release, that are critical for neutrophil mobilization from the BM during the onset of acute inflammation. Moreover, our results show that disruption of one HIF-1α allele is sufficient to impair lactate production and release by activated BM neutrophils and to reduce their mobilization to the circulation.

### Lactate increases BM endothelium permeability via GPR81

**Fig. 5:**
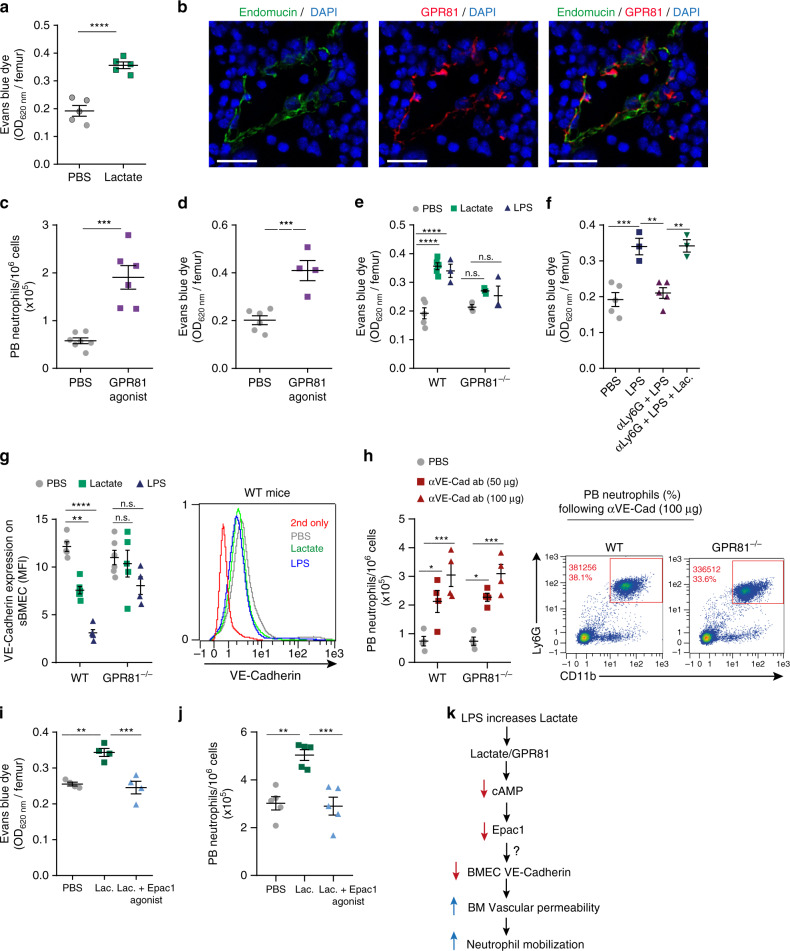
Lactate downregulates VE–cadherin and increases vascular permeability. **a** Levels of BM Evans Blue Dye (EBD) absorbance per femur at 620 nm in WT mice treated with PBS or lactate (*n* = 5). **b** Representative fluorescence images of Endomucin (green), GPR81 (red; RFP), and nuclei (blue; DAPI) in the femoral diaphysis of GRP81-RFP reporter mice; yellow indicates Endomucin^+^ GPR81^+^ sinusoidal BM endothelial cells. Representative images out of four independent experiments are shown. Scale bar indicates 20 μm. **c** PB neutrophils frequency following 4 h post administration of GPR81 agonist in WT mice (*n* = 7, PBS; *n* = 6, GPR81 agonist). ***p(0.0002). **d**–**f** Levels of BM EBD absorbance following GPR81 agonist treatment to WT mice (**d**, *n* = 6, PBS; *n* = 4, GPR81 agonist; ***p(0.0009)), after lactate or LPS injection to WT vs. GPR81^−/−^ mice (**e**, *n* = 5, WT + PBS or lactate; *n* = 3, WT + LPS; *n* = 3, GPR81^−/−^ + PBS; *n* = 4, GPR81^−/−^ + lactate; *n* = 3, GPR81^−/−^ + LPS) and following neutrophil depletion and exposure to LPS and lactate in WT mice (**f**, *n* = 5, PBS; *n* = 3, LPS; *n* = 5, α-Ly6G Abs + LPS; *n* = 3, α-Ly6G Abs + LPS + lactate). **g** Quantitative analysis and representative flow cytometry histogram plot of surface VE–cadherin expression on sBMEC 30 min post injection of lactate or LPS to WT vs. GPR81^−/−^ mice (WT; *n* = 5, PBS or LPS; *n* = 6, lactate; GPR81^−/−^; *n* = 6, PBS; *n* = 5, lactate; *n* = 4, LPS). **h** Quantitative analysis and representative flow cytometry density plots for PB neutrophils following administration of blocking anti-VE–cadherin antibodies to WT vs. GPR81^−/−^ mice (*n* = 4 per group). **i**, **j** Levels of BM EBD absorbance and neutrophil frequency in PB following treatment with lactate alone or with Epac1 agonist (**i**, *n* = 4 per group; **j**, *n* = 5 per group). **i** **p(PBS vs. lactate) = 0.0018; ***p(lactate vs. lactate+Epac1 agonist) = 0.0009; **j** **p(PBS vs. lactate) = 0.0013; ***p(lactate vs. lactate+Epac1 agonist) = 0.0008. **k** A scheme depicting lactate mode of action controlling BM vascular permeability via GPR81 signaling. Data are represented as mean ± SEM from 4 to 5 independent experiments. **p < 0.01; ***p < 0.001; ****p < 0.0001, Student’s two-tailed unpaired *t* test (**a**, **c**, **d**), one-way ANOVA with Tukey’s post hoc test (**f**, **i**, **j**) or two-way ANOVA with Tukey’s post hoc test (**e**, **g**, **h**). See also Supplementary Fig. 6.

Others and we have shown that BM sinusoidal vessels are more permeable and serve as the exclusive trafficking site for both immature hematopoietic stem cells and mature leukocytes^24,34–36^. To assess the involvement of BM sinusoidal “leakiness” in LPS- and lactate-induced neutrophil mobilization, we measured the penetration of Evans Blue Dye (EBD) from the circulation into the BM, a classical readout of BM vascular permeability. Indeed, lactate administration to WT mice increased BM vascular permeability within 4 h (Fig. 5a). Surprisingly, we identified that both sinusoidal (sBMECs; Endomucin^+^^ 37,38^, CD45^−^/CD31^+^/Sca-1^−^ ^24,38^) and arterial BM endothelial cells (aBMECs; Endomucin^−^ ^37,38^, CD45^−^/CD31^+^/Sca-1^+^ ^24^) express the lactate-receptor GPR81 (Fig. 5b, Supplementary Fig. 6a–c). To study the contribution of GPR81 signaling to BM vascular permeability and neutrophil mobilization, we treated mice with a specific GPR81 agonist (3,5-DHBA). This agonist reduced BM neutrophil numbers (Supplementary Fig. 6d) and increased their numbers in PB (Fig. 5c, Supplementary Fig. 6e) and liver (Supplementary Fig. 6f) 4 h after administration. The GPR81 agonist also directly enhanced BM vascular permeability, as reflected by EBD penetration to the BM in WT mice (Fig. 5d). Importantly, using GPR81^−/−^ mice we found that GPR81 is essential for enhanced BM vascular permeability induced by lactate or LPS (Fig. 5e). Strikingly, we observed that selective depletion of neutrophils resulted in a reduction in BM vascular permeability in mice exposed to LPS (Fig. 5f) which was rescued by exogenous lactate treatment. These results suggest that lactate produced and released from BM neutrophils in the course of LPS-induced inflammation directly elevates BM vascular permeability by binding and signaling through endothelial GPR81.

Previously it was shown that lactate activates GPR81 and suppresses cAMP production by reducing adenylyl cyclase activity^21^. cAMP regulates endothelial permeability through its direct binding to Exchange protein directly activated by cAMP (Epac)^39,40^, which is critical for the maintenance of vascular endothelial cadherin (VE–cadherin)-mediated adherence junctions^41,42^. Consistent with a role of lactate/GPR81 axis in enhanced vascular permeability, we found that lactate, LPS or GPR81 agonist preferentially reduced VE–cadherin expression by the more permeable sBMECs in WT mice compared to their GPR81^−/−^ counterparts (Fig. 5g, Supplementary Fig. 6g). Interestingly, this reduction was not observed on the less permeable aBMECs that express higher levels of surface VE–cadherin to begin with^24^ (Supplementary Fig. 6h).

Notably, enforced disruption of VE–cadherin assemblies in GPR81^−/−^ mice with a VE–cadherin-function blocking antibody that impairs vascular integrity^41^, elevated PB neutrophil levels, similar to WT mice (Fig. 5h). These results further suggest that VE–cadherin expression and activity in the BM vasculature is modulated by GPR81 signaling triggered by lactate.

To determine if lactate induces the vascular permeability changes necessary for optimal BM neutrophil mobilization via the cAMP/Epac1 axis, we co-injected WT mice with lactate alone or in combination with a selective Epac1 agonist^40^. We found that Epac1 agonist reduced lactate-induced BM vascular permeability (Fig. 5i) and neutrophil mobilization to the circulation (Fig. 5j, Supplementary Fig. 6i). Altogether, our results indicate lactate-driven activation of GPR81 signaling in sBMEC and suggest a downstream reduction in cAMP and Epac1 activation. This may be the mechanism that reduces VE–cadherin expression leading to increased BM vascular permeability and enhanced neutrophil mobilization during acute inflammation (Fig. 5k).

### GPR81/lactate signaling mediates neutrophil mobilization

**Fig. 6:**
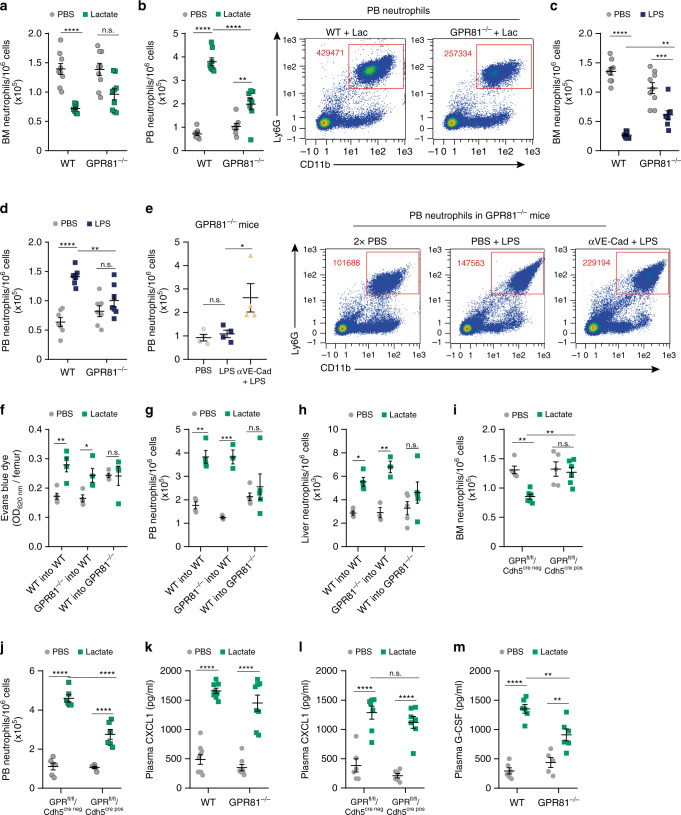
GPR81/lactate signaling mediates neutrophil mobilization. **a**, **b** Frequency of neutrophils in the BM (**a**, *n* = 9) and quantitative analysis and representative flow cytometry density plots for PB neutrophils (**b**, *n* = 8) following lactate treatment in WT vs. GPR81^−/−^ mice. **p(GPR81^−/−^ + PBS vs. GPR81^−/−^ + lactate) =0.0017. **c** Frequency of BM neutrophils in WT (*n* = 9, PBS; *n* = 8, LPS) vs. GPR81^−/−^ (*n* = 9, PBS; *n* = 8, LPS) mice following LPS treatment. **d** Frequency of PB neutrophils following LPS administration (*n* = 7). **e** Quantitative analysis and representative flow cytometry density plots for PB neutrophils following blocking VE–cadherin antibodies in GPR81^−/−^ mice (*n* = 4). *p(LPS vs. αVE–Cad + LPS) = 0.0402. **f** Levels of BM EBD absorbance following lactate treatment in chimeric mice (*n* = 5, WT to WT + PBS; *n* = 4, WT to WT + lactate; *n* = 4, GPR81^−/−^ to WT; *n* = 4, WT to GPR81^−/−^). **g**, **h** Neutrophil frequency in the PB (**g**, *n* = 4, WT to WT; *n* = 3, GPR81^−/−^ to WT; *n* = 5, WT to GPR81^−/−^), and in the liver of chimeric mice (**h**, *n* = 4, WT to WT; *n* = 3, GPR81^−/−^ to WT; *n* = 5, WT to GPR81^−/−^) following lactate administration. **i**, **j** Frequency of neutrophils in the BM (**i**; *n* = 5, PBS; *n* = 6, lactate) and blood (**j**, *n* = 6) following lactate treatment in GPR81^f/f/^Cdh5^cre neg^ vs. GPR81^f/f^/Cdh5^cre pos^ mice. **k**, **l** Plasma protein levels of CXCL1 in **k** WT vs. GPR81^−/−^ mice (WT; *n* = 9, PBS; *n* = 8, lactate; GPR81^−/−^; *n* = 8) or **l** GPR81^f/f/^Cdh5^cre neg^ vs. GPR81^f/f^/Cdh5^cre pos^ mice (*n* = 6, PBS; *n* = 7, lactate; per mice group) following lactate treatment. **m** Plasma protein levels of G-CSF in WT vs. GPR81^−/−^ mice (*n* = 6, WT + PBS, WT + lactate and GPR81^−/−^ + lactate; *n* = 5 GPR81^−/−^ + PBS) following lactate treatment. Data are represented as mean ± SEM from 3 to 5 independent experiments. *p < 0.05; **p < 0.01; ***p < 0.001; ****p < 0.0001, two-way ANOVA with Tukey’s post hoc test (**a**–**d**, **f**–**m**) or one-way ANOVA with Tukey’s post hoc test (**e**). See also Supplementary Fig. 7.

Next, we investigated the impact of lactate/GPR81 signaling on neutrophil mobilization. Pharmacological inhibition of GPR81 signaling^43^ markedly reduced the effect of lactate on neutrophil mobilization from the BM to the PB and recruitment to the liver (Supplementary Fig. 7a–c; respectively). Furthermore, a significant lactate-induced reduction in BM neutrophil levels was observed in WT but not in GPR81^−/−^ mice (Fig. 6a), coupled with a large increase in neutrophil numbers in PB and liver of WT mice and a smaller but significant increase in the numbers of PB mobilized neutrophils in GPR81^−/−^ mice treated with lactate (Fig. 6b, Supplementary Fig. 7d). Furthermore, LPS-induced mobilization of BM neutrophils was also lower in GPR81^−/−^ mice compared to their WT counterparts (Fig. 6c, d, Supplementary Fig. 7e). Importantly, this attenuated neutrophil mobilization following LPS treatment in GPR81^−/−^ mice was rescued by administration of blocking VE–cadherin antibodies^41^ (Fig. 6e). This finding implicates LPS as a potent regulator of VE–cadherin dependent BM vascular permeability via its ability to trigger lactate release.

To determine the relative contributions of vascular, stromal and hematopoietic GPR81 signaling (Supplementary Fig. 1f, Supplementary Fig. 3d^44–46^) to vascular permeability and neutrophil mobilization induced by lactate we generated chimeric mice in which hematopoietic cells from the BM of WT mice were transplanted into GPR81^−/−^ mice and vice versa (Supplementary Fig. 7f). The effects of lactate treatment to these chimeric mice demonstrated that lactate/GPR81 signaling in BM endothelial and stromal cells, but not in hematopoietic cells, control the increase in BM vascular permeability and neutrophil mobilization (Fig. 6f–h). Moreover, to better reveal the specific function of endothelial lactate/GPR81 signaling in neutrophil mobilization, we generated inducible endothelial-specific GPR81 KO mice. We observed a significant lactate-induced reduction in BM neutrophil levels in GPR81^flox/flox^/Cdh5^cre neg^ (WT counterparts) but not in endothelial-specific GPR81 KO mice (GPR81^flox/flox^/Cdh5^cre pos^; Fig. 6i), coupled with a large increase in PB neutrophil levels of WT counterparts and a smaller but significant increase in PB mobilized neutrophils in endothelial-specific GPR81 KO mice treated with lactate (Fig. 6j).

The residual lactate-induced neutrophil mobilization we detected in both GPR81^−/−^ and in inducible endothelial-specific GPR81 KO mice (Fig. 6b, j) raised the possibility that lactate triggers additional pathways independent of GPR81 signaling which promote neutrophil mobilization from the BM. Since the chemokines CXCL1 and CXCL2^47–51^ and the neutrophil-mobilizing cytokine G-CSF^51–53^ contribute to the early stages of neutrophil mobilization and recruitment during acute inflammation, we next examined the potential effects of lactate concerning the release of these factors in BM and blood. Notably, we found that lactate treatment dramatically increased CXCL1 levels in both compartments in an endothelial-GPR81-independent manner (Supplementary Fig. 7g, h, Fig. 6k, l). We also observed a more moderate increase in the levels of CXCL2 in both BM and blood of WT mice treated with lactate (Supplementary Fig. 7i, j). Lactate-induced elevation of CXCL1 and CXCL2 most probably downregulated surface expression of their receptor CXCR2 by PB neutrophils (Supplementary Fig. 7k). Importantly, we also found that lactate administration dramatically increased blood G-CSF levels in WT mice and a smaller but significant increase in GPR81^−/−^ (Fig. 6m). These results could explain the residual lactate-induced neutrophil mobilization we observed in the two models of GPR81^−/−^ mice and why only neutrophils are rapidly mobilized 4 h post lactate administration despite the increased BM vascular permeability. Collectively our results reveal that the lactate/GPR81 axis is a key regulator of BM vascular permeability required for optimal mobilization of BM neutrophils to the blood and their recruitment to inflamed organs. In addition to this axis, lactate also triggers the release of neutrophil mobilizing chemokines and G-CSF, key inducers of rapid neutrophil mobilization from the BM.

### Salmonella increases lactate production by BM neutrophils

**Fig. 7:**
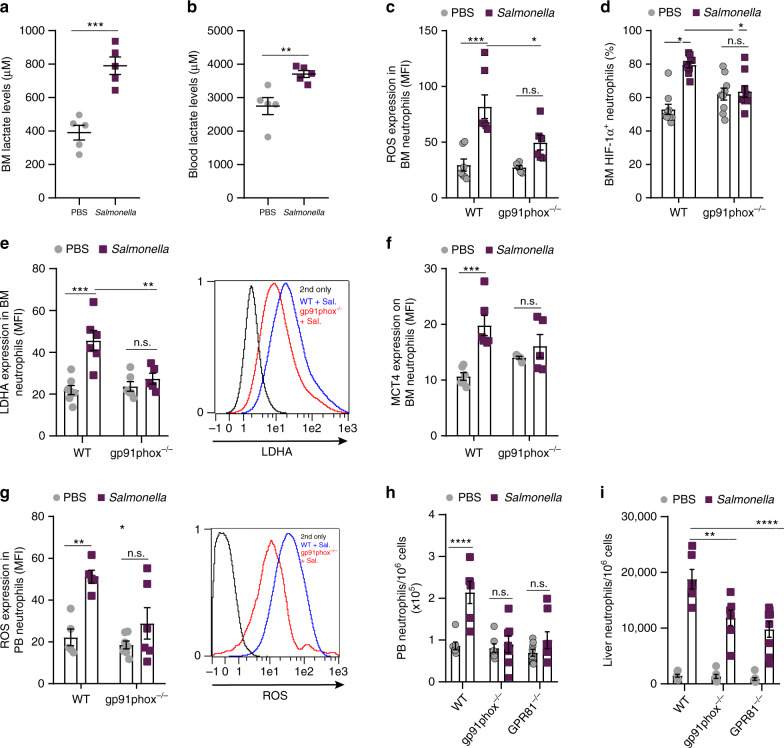
*Salmonella* increases lactate production by inflammatory BM neutrophils. **a**, **b** Lactate levels in (**a**) BM or (**b**) blood from WT mice treated with PBS or *Salmonella* (*n* = 5). **a** ***p(0.0004); **b** **p(0.0081). **c** Quantitative analysis of ROS production in BM neutrophils from WT (*n* = 7) vs. gp91phox^−/−^ (*n* = 6) mice infected with *Salmonella*. **d** Percentage of BM HIF-1α^+^ neutrophils in WT (*n* = 9) vs. gp91phox^−/−^ (*n* = 9). **e** Quantitative analysis and representative flow cytometry histogram plot of LDHA expression in BM neutrophils from WT (*n* = 7, PBS; *n* = 6, *Salmonella;* ***p(0.0001)) vs. gp91phox^−/−^ (*n* = 6, PBS; *n* = 5, *Salmonella*) mice. **f** Quantitative analysis of MCT4 expression on BM neutrophils from WT (*n* = 6; ***p(0.0008)) and gp91phox^−/−^ (*n* = 5) mice. **g** Quantitative analysis and representative flow cytometry histogram plot of ROS production in PB neutrophils from WT (*n* = 5; **p(0.0030)) vs. gp91phox^−/−^ (*n* = 7, PBS; *n* = 6, *Salmonella*) mice. **h** Frequency of PB neutrophils from WT (*n* = 7, PBS; *n* = 6, *Salmonella*) vs. gp91phox^−/−^ (*n* = 7) and GPR81^−/−^ (*n* = 8) mice. **i** Frequency of liver neutrophils from WT (*n* = 7, PBS; *n* = 6, *Salmonella)* vs. gp91phox^−/−^ (*n* = 7) and GPR81^−/−^ (*n* = 8) mice. Data are represented as mean ± SEM from three independent experiments. *p < 0.05; **p < 0.01; ***p < 0.001, ****p < 0.0001, Student’s two-tailed unpaired *t* test (**a**, **b**), two-way ANOVA with Tukey’s post hoc test (**c**–**i**).

Finally, to examine the clinical relevance of our findings, we infected mice with gram-negative *Salmonella*Typhimurium and found higher levels of lactate in both BM and blood of WT mice (Fig. 7a, b; respectively), similar to those prompted by LPS (Fig. 1f, Supplementary Fig. 1j). Next, we infected gp91phox^−/−^ mice and compared their metabolic parameters with WT mice. Our findings indicate that following the onset of infection (4 h after bacterial challenge) WT BM neutrophils displayed high generation of ROS, elevated levels of HIF-1α^+^ neutrophils as well as lactate production and release (Fig. 7c–f; respectively). In contrast, similar infection of gp91phox^−/−^ mice did not increase the numbers of BM HIF-1α^+^ neutrophils as observed in WT counterparts (Fig. 7d). Interestingly, the deficiency in NOX/ROS activities in infected gp91phox^−/−^ mice resulted in impaired rates of lactate production and release from BM neutrophils infected with *Salmonella* (Fig. 7e, f). Consequently, WT mice infected with *Salmonella* also have higher levels of activated neutrophils in the blood and liver, as compared to gp91phox^−/−^ or GPR81^−/−^ mice counterparts (Fig. 7g–i). Of note, although to a lower extent than in WT mice, elevated levels of liver neutrophils were also observed in *Salmonella-*infected gp91phox^−/−^ or GPR81^−/−^ mice, suggesting that the NOX and GPR81 axes are major but not exclusive players in *Salmonella*-induced neutrophil recruitment.

Altogether, our data reveals that the regulatory mechanisms identified in the present work by which neutrophils respond to enforced LPS challenges are also activated during physiological bacterial infections with *Salmonella*, demonstrating its clinical relevance.

## Discussion

**Fig. 8:**
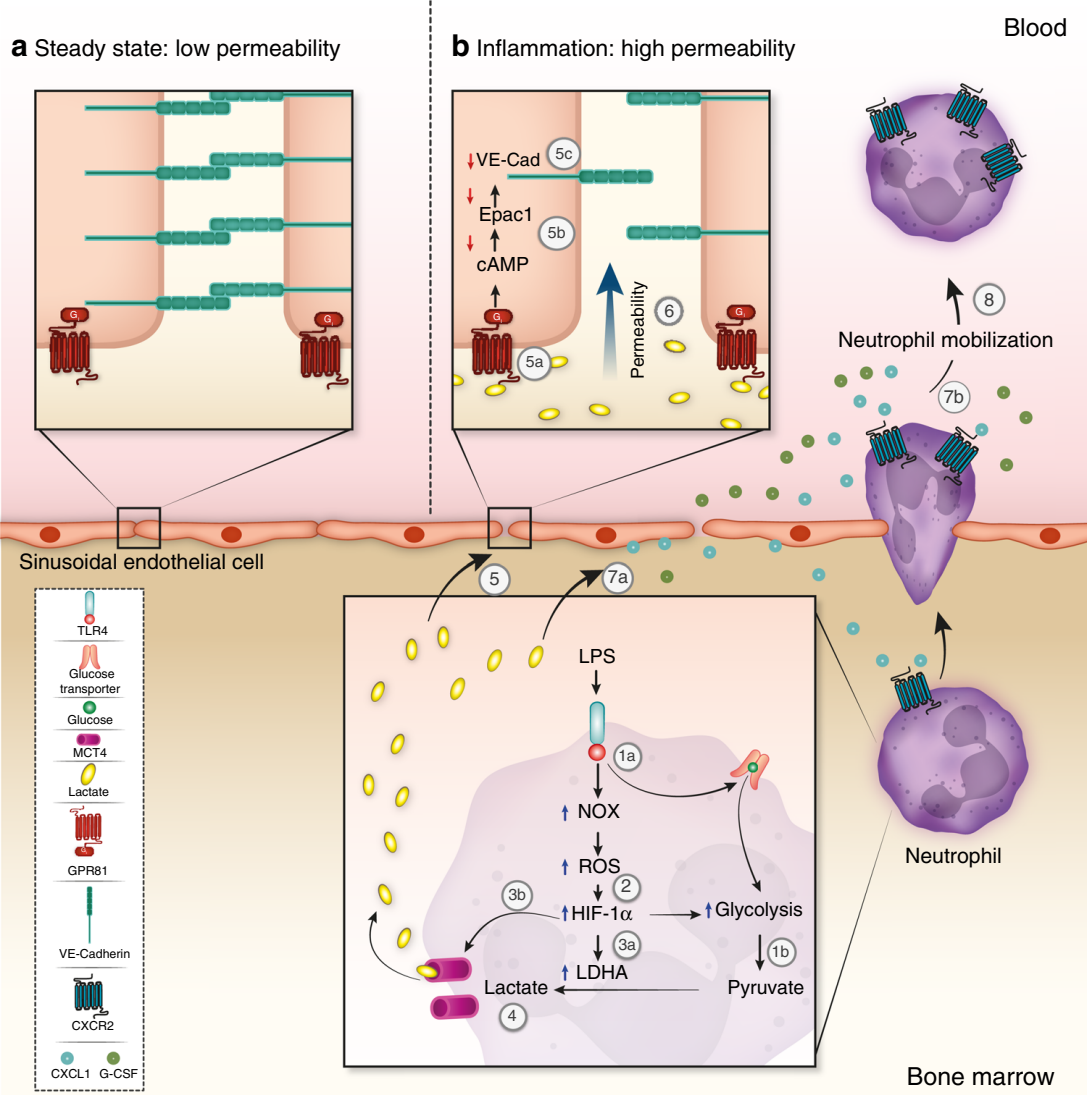
Mechanisms of lactate-induced neutrophil mobilization from the BM. Our model suggests that enhanced lactate-produced by BM neutrophils during bacterial infection induces neutrophil mobilization by modulating metabolic signaling in BM endothelial cells. LPS binds TLR4 expressed on neutrophils that directly activates NADPH oxidase (NOX) and enhances glucose uptake via a glucose transporter 1a. Glucose in turn, is converted to pyruvate by the glycolysis pathway 1b. NOX activity leads to ROS production (2) which elevates HIF-1α expression. HIF-1α in turn, induces downstream expression of LDHA that converts pyruvate to lactate 3a. HIF-1α also up-regulates the lactate transporter MCT4 3b to allow lactate release (4). Under steady-state conditions, surface VE–cadherin on endothelial cells (ECs) is highly expressed, which maintains the endothelial barrier integrity with low permeability (**a**). During inflammation (**b**), lactate released from BM neutrophils binds to GPR81 on sinusoidal BM endothelial cells 5a, activates G_i_ protein and thereby reduces cAMP/Epac1 activity 5b. Consequently, lactate via GPR81 signaling decreases surface VE–cadherin expression (5c), leading to higher BM vascular permeability (6). In addition, lactate elevates CXCL1 and G-CSF levels (produced by different cells including ECs^50,52^) also in a GPR81-independent manner 7a with more moderate increases in CXCL2 levels in WT mice (most probably produced by BM neutrophils^50^). Lactate-induced elevation of CXCL1, CXCL2, and G-CSF downregulates surface CXCR2 expression on PB neutrophils 7b facilitating neutrophil mobilization (8). Taken together, LPS-induced lactate promotes rapid neutrophil mobilization from the BM to the blood (8) preferentially via BM GPR81/VE–cadherin-dependent (5–6) and also by GPR81-independent (7) pathways.

In this study, we describe a role for the metabolite lactate implicated in the early phase of acute inflammation triggered by bacterial LPS. Our findings reveal that lactate acts as a critical regulator of rapid neutrophil mobilization from the BM to the circulation induced by LPS. We also found that LPS-induced inflammation enhances glycolytic activities in BM neutrophils that promote lactate production and release. Lactate released from inflammatory BM neutrophils triggers their mobilization acting on its receptor GPR81 functionally expressed by endothelial cells to locally increase vascular permeability. In addition, lactate facilitates rapid neutrophil mobilization from the BM also by elevating the levels of neutrophil-attracting chemokines CXCL1 and CXCL2, and by increasing the release of the cytokine G-CSF. A schematic model summarizing these processes is presented in Fig. 8.

Lactate is not only a waste byproduct of cell metabolism that merely accumulates at inflammatory sites^54^ and at the tumor microenvironment, but instead is an effector molecule that contributes to inflammation and to both, onset and progression of cancer^55^. Recently, lactate was also identified as an effector metabolite that triggers multiple cell signaling pathways in various organs^21,22,56,57^. Furthermore, the role of lactate in regulation of immune cells is documented in different diseases, including cancer and multiple sclerosis^55,58–60^. Hence, inhibitors to lactate dehydrogenase and MCTs could be effective targets to attenuate undesirable neutrophil mobilization in pathological inflammatory processes and potentially also in malignancies facilitated by tumor helper neutrophils^61^. Since lactate can also boost TLR4 activation and transcription of pro-inflammatory genes through NF-κB signaling in innate immune cells like macrophages^18^, such inhibitors may be useful in controlling pathologies resulting in excess activation of these subsets.

Importantly, elevated lactate levels coinciding with the rise of neutrophils in the circulation and inflamed tissues are associated with pathological conditions including shock, sepsis, and ischemia^62^. Lactate was also found to be released by human neutrophils^12^ and it was shown that glucose uptake, enhanced glycolysis and lactate promoted neutrophil functions like phagocytic activity^63^ and neutrophil extracellular traps formation^64,65^. However, how neutrophils produce lactate, and how this metabolite affects neutrophil function and mobilization during acute inflammation have been open questions. Our results directly link lactate production by BM neutrophils to ROS generation by the phagocytic NOX machinery and activation of HIF-1α. Indeed, defects in this inflammatory metabolic pathway were found in neutrophils derived from NOX^−/−^ (gp91phox^−/−^) mice treated with LPS or infected with *Salmonella*. In addition, NOX^−/−^ mice treated with LPS mobilized reduced levels of neutrophils in general and activated ROS^high^ neutrophils in particular. Importantly, lactate treatment preferentially restored the reduced levels of mobilized ROS^high^ neutrophils in the blood of NOX^−/−^ mice exposed to LPS.

Distinct metabolic pathways play pivotal roles in immune cell regulation and function. In particular, HIF-1α controls key glycolytic programs in many cell types and different hypoxic environments^32,33,66^. However, LPS can promote glycolysis and induce HIF-1α expression in macrophages even under non-hypoxic conditions. We report that LPS increased glucose uptake and triggered the activities of glycolytic enzymes in BM neutrophils via HIF-1α elevation. Indeed, using mice with a myeloid-specific defect in HIF-1α, we found that this transcription factor is critical for glycolysis and plays essential roles in lactate production and release by BM neutrophils during acute inflammatory responses, key checkpoints in neutrophil mobilization from the BM. Interestingly, in support of our results, intense exercise was reported to elevate plasma lactate levels and to enhance neutrophil–endothelial cell interactions in the muscle microvasculature in a ROS-dependent manner^66^. However, in that study lactate was not confirmed to act as a direct mediator of neutrophil–endothelial interactions. In addition, a recent study reported that facilitating the crosstalk of neutrophils with endothelial cells improves blood vessel regeneration, and hematopoietic recovery^67^. Our results demonstrate that during the early phase of acute inflammation, LPS activates BM neutrophils to produce and release lactate to the BM microenvironment. Lactate within the BM communicates with BM-blood endothelial vessels to open the junctions for neutrophil mobilization and recruitment through GPR81 signaling, to distant infected organs. LPS can mobilize neutrophils via direct and indirect mechanisms, therefore, we cannot rule out that the effects of LPS challenge may also involve other BM cell types which functionally express TLR4.

Previously, the neurotransmitter neuropeptide Y (NPY) activation in endothelial cells (ECs) was found to reduce VE–cadherin expression along ECs junctions, resulting in increased vascular permeability and hematopoietic stem and progenitor cell (HSPC) mobilization^42^. Along these lines, we found that mechanistically, lactate signaling via its endothelial GPR81 receptor decreased VE–cadherin expression preferentially by the more permeable sinusoidal BM ECs, which enhances BM vascular permeability and neutrophil mobilization from the BM. Properties of the endothelial barrier are normally maintained by high levels of cAMP. This secondary messenger activates Epac1 and its main downstream GTPase, Rap-1 reported to stabilize VE–cadherin assemblies, which is critical for low vascular permeability. Endothelial barrier disruption by inflammatory stimuli such as bacterial LPS^68^ or TNF-α^69^ is partially associated with decreased cAMP and Epac1 activity as well as reduced VE–cadherin expression. These mechanisms may act in concert with the lactate-triggered GPR81 signaling axis found by us to acquire the threshold endothelial permeability, critical for neutrophil mobilization from the BM^24,34^.

G-CSF is a major neutrophil-mobilizing cytokine under both basal and stress conditions^2,51,70,71^. G-CSF is also a hematopoietic cytokine, produced by different BM cell types, including ECs^52^, osteoblasts^72^, and osteocytes^73^. In addition, G-CSF has multiple functions including regulation of neutrophil progenitor cell proliferation and differentiation^52^ and the functional activation of neutrophils^74^. Several other neutrophil-mobilizing agents are responsible for rapid neutrophil mobilization during the early stage of acute inflammation, the most notable being CXCR2 ligands (CXCL1 and CXCL2^51,75^). At peripheral sites of inflammation CXCL1 produced by inflamed ECs can also mediate neutrophil adhesion and intraluminal crawling, whereas CXCL2-produced by neutrophils is implicated in neutrophil breaching of endothelial-cells junctions^50^. In addition to its direct effect on BM vascular permeability, lactate acts dramatically on BM neutrophil rapid mobilization by elevating the levels of circulating G-CSF and the chemokines CXCL1 and CXCL2, leading to downregulation of CXCR2 expression on blood neutrophils. A recent study^76^ reported that G-CSF produced in response to *Escherichia coli*-challenge, inhibits CXCR2-mediated cellular signaling. The reduction in CXCR2 expression we observed in our model following lactate treatment can therefore, stem from both internalization induced by CXCL1/2 upregulation as well as from G-CSF induced inhibition. Importantly, lactate elevated CXCL1 levels independently of GPR81 signaling and increased G-CSF in both GPR81-dependent and -independent manners. These results may explain the residual lactate-induced neutrophil mobilization we observed in GPR81^−/−^ mice and why only neutrophils are rapidly mobilized 4 h following lactate treatment despite the increased BM vascular permeability. In light of our results, we hypothesize that lactate induces the release of CXCL1, CXCL2, and G-CSF preferentially via its influx transporter MCT1. In support of our results, a recent study showed that in addition to chemokines release, increased lung vascular permeability can also impact neutrophil trafficking^77^. Our model indicates that lactate acts as a pro-inflammatory factor that enhances neutrophil mobilization via increasing BM vascular permeability and elevating chemokines/G-CSF that are pivotal features of an acute inflammatory response.

In addition to lactate, neutrophils release additional effector molecules such as proteolytic enzymes to facilitate their breaching of endothelial junctions^78,79^. Our study is, however, the first example of a neutrophil-released metabolite, which is a potential target for modulating neutrophil mobilization to peripheral organs exposed to acute infections. Our findings should also prompt an exploration for additional metabolites potentially released by other leukocyte subsets with homologous ability to modulate their barrier crossing under various inflammatory stresses.

## Methods

### Mice

C57BL/6 mice were purchased from Harlan Laboratories (Rehovot, Israel). loxP-flanked HIF-1α (B6.129- Hif1atm3Rsjo/J) mice, LysM-Cre (B6.129P2-Lyz2tm1(cre)Ifo/J) mice, gp91phox^−/−^ (B6.129S-Cybbtm1Din/J) mice and transgenic Ly6a(Sca-1)-eGFP mice were purchased from Jackson Laboratories. Transgenic LysM-GFP mice were kindly provided by G. Shachar (Weizmann Institute, Israel). GPR81^−/−^ and GPR81-RFP mice were kindly provided by S. Offermanns (Department of Pharmacology, Max-Planck-Institute for Heart and Lung Research, Germany). Catchup (Ly6G tdTomato) mice were kindly provided by M. Gunzer (University Duisburg-Essen, Germany). Conditional mutants carrying loxP-flanked GPR81 were kindly provided by Z. Gerhart-Hines (University of Copenhagen, Denmark). To induce endothelial-specific Cre activity and GPR81 inactivation/expression, adult VE–cadherin (Cdh5, PAC)-CreERT2 mice^80^ were interbred with GPR81^flox/flox^ (conducted in the lab of S. Offermanns; Max-Planck-Institute for Heart and Lung Research, Germany). Mice were injected intraperitoneally (i.p.) with Tamoxifen (Sigma, T5648) at 1 mg per mouse per day for 5 days. Mice were allowed to recover for 3 weeks after tamoxifen injections, before experimental analysis. Mice carrying only GPR81^flox/flox^ mutation (Cdh5 cre negative) were used as WT controls. Primers used to test the presence of the *Cdh5-CreERT2* recombinase gene were: 5′-ACTGGGTCCTGATGGTGCC-3′ and 5′-GTGAAACAGCATTGCTGTCACTT-3′. To introduce a dysfunctional HIF-1α in a myeloid-specific lineage, we crossed mice containing loxP sites flanking exon 2 of HIF1α with LysM-Cre mice. All mouse offspring from all strains were routinely genotyped using polymerase chain reaction analysis of DNA from tail biopsies. Primers used (for *HIF-1α flox/LysM Cre*) to distinguish floxed from non-floxed alleles were: 5′-GCA GTT AAG AGC ACT AGT TG-3′ and 5′-GGA GCT ATC TCT CTA GAC C-3′ for *HIF-1α*^81^. Primers used to test the presence or absence of the *LyzM Cre* recombinase gene were: 5′CRE (TGCAAGTTGAATAACCGGAAA) and 3′CRE (CTAGAGCCTGTTTTGCACGTTC).

Breeding and all experimental procedures were monitored by the Veterinary Resources Unit of the Weizmann Institute and were approved by the Institute Animal Care and Use Committee (IACUC). All mutated or transgenic mouse strains were compared to strain-matched WT C57BL/6 sex-and age-matched controls. Totally, 8–10-week-old mice, both male, and female were used for all experiments. BM cells were obtained by flushing long bones with phosphate-buffered saline (PBS), and peripheral blood was collected from the heart using heparinized syringes. No blinding was used to allocate experimental groups.

### In vivo treatments

Sodium-Lactate (Sigma) was injected intraperitoneally (i.p.; 30 or 50.5 mg dissolved in 200 μl PBS, pH‐adjusted to ~7.0) 4 h or 30 min before mice sacrifice and cells harvest. These lactate doses are non-lethal and do not cause acidosis (Table 1) or severe inflammatory side effects in strains applied for this study^21,55,82^. In order to induce acute inflammation, LPS (from Escherichia coli 0111:B4, L2630, Sigma) was injected i.p. (at 50 µg per injection^83^) 4 h or 30 min before cells were harvested. This LPS dose is non-lethal and does not cause acidosis (Table 1) in strains used for this study.

LDHA inhibitors; Sodium oxamate (Sigma, dissolved in PBS, 750 mg/kg) or FX11 (selective, reversible, NADH competitive LDHA, Calbiochem, dissolved in DMSO, 42 μg/mouse) and MCT4 inhibitor CHC (2-Cyano-3-(4-hydroxyphenyl)-2-propenoic acid, TOCRIS, dissolved in DMSO, 40 μM/mouse) were injected i.p. three times: at 7AM together with LPS, and two additional injections of LDHA inhibitor or MCT4 inhibitor 15 and 30 min following the first injection, to inhibit LDHA or MCT4 activity. PBS or PBS with DMSO served as vehicle control. All the mice received the same number of injections.

We noticed that the number of vehicle injections affects the levels and phenotype of circulating neutrophils. For example, experiments that included four injections of the vehicles PBS or PBS with DMSO, like in Fig. 2. These extra injections dramatically increased the number of total PB neutrophils compared to a single PBS injection. We observed two clear populations of PB neutrophils (CD11b^int^/Ly6G^high^ and CD11b^high^/Ly6G^high^) following PBS injections but only the CD11b^high^/Ly6G^high^ population was affected by LPS (increased to 15% and decreased in the presence of the inhibitors; Fig. 2h–k). The CD11b^int^/Ly6G^high^ population, which increased only after PBS injections (without elevation of CD11b expression, a marker for primed and activated neutrophils following bacterial signals) disappeared after LPS treatment.

GPR81 antagonist (3-hydroxy-butyrate; 3-OBA, Sigma, dissolved in PBS) was injected i.p. at 15 mM/mouse three times, at 7 A.M. together with lactate and two additional injections were performed 15 and 30 min following the first injection, to inhibit GPR81 signaling. GPR81 agonist (3,5-dihydroxybenzoic acid; 3,5-DHBA, TOCRIS, dissolved in PBS) was injected i.p. at 30 mg/kg 4 h or 30 min before cells were harvested. Epac1 agonist (8-CPT-2′-O-Me-cAMP; TOCRIS, dissolved in PBS) was injected i.p. at 1 mM/mouse twice, at 7 A.M. together with lactate and one additional injection was performed 1 h following the first injection, to inhibit the BM vascular permeability.

For quantifying the number of liver neutrophils, a similar piece of the liver (size-length: 1.7 × width: 1.2 cm) from the same lobe was taken from each mouse.

### Neutrophil depletion

Totally, 125 μg purified anti-Ly6G antibody^67^ (clone 1A8, Bio X Cell) diluted in 200 μl PBS was administered i.p. at 24 h prior to LPS injection. All the mice were sacrificed at 11 A.M.

Gr-1 and CD11b antibodies were used for BM neutrophils detection. Frequency of BM FSC^high^/SSC^high^ cells reduction was observed following Ly6G depletion (Supplementary Fig. 1c). In addition, depletion of ~85% of BM neutrophils (Gr-1^high^/CD11b^high^) was also observed following depletion (Supplementary Fig. 1c).

### Blood pH measurement

Measurements of blood pH and glucose levels in WT mice following PBS, lactate or LPS treatment, were performed by using i-STAT clinical analyzer (Abaxis). Peripheral blood was collected from the heart using syringes coated with lithium heparin. For the i-STAT analysis 100 μl of blood was immediately placed into a cartridge (i-STAT CG8, PET-VETBIOMED) to determine the parameters following the manufacturer’s instructions. The i-STAT machine was calibrated using the Electronic Simulation module before the analysis of samples. Blood glucose levels were used as a control for the quality, and reliability of the machine (test). Both lactate and LPS treatments reduced the blood levels of metabolic glucose without affecting the blood pH (Table 1).

### Endothelial VE–cadherin disruption

Purified blocking anti-VE–cadherin antibodies (50 or 100 μg as indicated, clone BV13, functional Grade, Invitrogen) was administered intravenously (i.v.) to WT or GPR81^−/−^ mice 4 h before cells harvest in order to disrupt VE–cadherin homotypic adhesion, clustering and endothelial integrity.

For rescue experiments (Fig. 6e) 50 μg of purified anti-VE–cadherin antibodies were administered 1 h (at 6:00 A.M.) before LPS injection (at 7:00 A.M.). All the mice were sacrificed at 11:00 A.M. PBS alone served as a vehicle control.

### Neutrophil isolation

Flushed BM cells (from WT and mutated mice) were resuspended in 200 μl MACS buffer (2 mM EDTA, 0.5% bovine serum albumin in PBS) and BM neutrophils were isolated according to the protocol of “Neutrophil isolation kit, mouse” by Miltenyi. The yields were approximately 8 × 10^6^ cells in total (for 6 bones of two legs), and the purity was >94%.

### In vitro treatment for lactate production

BM isolated neutrophils (1 × 10^6^) were seeded on 24-well plate in serum-free RPMI-1640 culture media and then stimulated with either PBS or 120 ng/ml LPS for 5 h. Cell-free supernatants from non-stimulated or LPS-stimulated cultured neutrophils were harvested at 5 h and analyzed for lactate content using an l-lactate assay (700510, Cayman).

### Flow cytometry

Cell populations were analyzed with MACSQuant (models 10 and VYB, Miltenyi, Bergisch Gladbach Germany), data were analyzed with MACSQant software. Cellular expression levels were analyzed and presented as MFI (mean fluorescent intensity). Isolated peripheral blood and liver cells underwent red blood cell lysis (R7577, Sigma) before staining. Flushed BM, peripheral blood and liver cells were stained for 30 min at 4 °C in flow cytometry buffer (PBS, 10% fetal bovine serum and 0.02% azide). For intracellular antigens staining (HIF-1α and LDHA), cell surface staining was followed by cell fixation and permeabilization with the Cytofix/Cytoperm kit (BD Biosciences) according to the manufacturer’s instructions. For neutrophil staining, we used anti-Ly6G-APC (A18, Biogems) and anti-CD11b-FITC (M1/70, BioLegend). For detection of depleted neutrophils, we used GR-1-APC (RB6-8C5, BioLegend). For sorting neutrophils, we used a combination of CD45-PE (30-F11, 103106)/Ly6G-APC/CD11b-FITC (all from BioLegend). For monocytes/macrophages staining, we used anti-Ly6C-PE-Cy7 and anti-CD11b-FITC (BioLegend). For lymphocytes staining, we used anti-CD4-FITC (GK15, 103106) and anti-B220-PE (RA3-6B2, 103208) (all from BioLegend). For intracellular ROS detection, cells were incubated for 10 min at 37 °C with 2 μM hydroethidine (Life Technologies) prior to cell surface staining for neutrophil markers as described above. For glucose uptake detection, cells were incubated for 30 min at 37 °C with the glucose analog 2-NBDG (Life Technologies). Staining without the addition of the ROS or 2-NBDG probe was considered the baseline for gating the positive population. For MCT4 expression, we used rabbit anti-mouse MCT4 (H-90, Santa Cruz) followed by anti-rabbit PE (catalog number: 711-116-152, Jackson ImmunoResearch). Data presenting MCT4 staining were obtained using two different flow cytometer analyzers, with different settings. Quantitative analyses presented in Figs. 1i, 3c, and Supplementary Fig. 5c were obtained using MACSQuant analyzer10, while Fig. 4c and Supplementary Fig. 4d were obtained using MACSQuant VYB. For MCT1 expression, we stained with rabbit anti-mouse MCT1 (M-45, Santa Cruz, sc-50325, B2316) followed by anti-rabbit PE (Jackson ImmunoResearch). HIF-1α was identified using conjugated anti-h/m HIF-1α-PE (241812, R&D). For LDHA expression, we stained with rabbit anti-mouse LDHA (EPR1564, Abcam) followed by anti-rabbit PE (Jackson ImmunoResearch). Data presenting LDHA staining were obtained using two different flow cytometer analyzers, with different settings. Quantitative analyses presented in Figs. 1e, 3b, and Supplementary Fig. 5b were obtained using MACSQuant analyzer10, while Figs. 4b, 7e, and Supplementary Fig. 4c were obtained using MACSQuant VYB. For BM endothelial cell staining, BM cells were flushed with Liver digest medium (LDM; Invitrogen) supplemented with 0.01% DNase 1 (Roche) and mechanically crushed by mortar and pestle in the same medium, followed by 30 min digestion at 37 °C with shaking. Following incubation time, cells were washed twice with PBS. Next, cells underwent red blood cell lysis (R7577, Sigma), filtered and washed extensively before staining. Sinusoidal or arterials endothelial cells were identified by staining with anti-CD45-APC, anti-CD31-PE-Cy7, and anti-Sca-1-PE or Pacific Blue (all from BioLegend) as described^24^. For GPR81 expression, we stained with rabbit anti-mouse GPR81 (Novus, catalog number NLS2095) followed by anti-rabbit PE (Jackson ImmunoResearch). For VE–cadherin expression, we stained with anti-VE–cadherin-BV421 conjugated antibody (11D4.1, BD Bioscience).

For FACS staining, we used the fluorescence minus one (FMO) as a negative control, which contains all the fluorochromes except for the one that is being measured. The FMO in the manuscript is presented as “secondary only”.

### BM cell sorting

BM single-cell suspensions were sorted gating on CD45^+^/CD11b^+/^Ly6G^high^ cells using SORP-FACSAriaII machine with a 100 μm nozzle. Dead cells were excluded based on DAPI incorporation reaching purities greater than 94%.

Cells (9000–35,000 cells per sample) were sorted into Eppendorf tubes containing 100 μl lysis buffer (RLT buffer (QIAGEN, 79216) with 1% 2-Mercaptoethanol). After sorting, lysis buffer was added to the samples to reach 2–4 times of the volume of the sorted cells. Cell lysis was achieved by vortex for 30 s. Samples were snap-frozen on dry ice and stored at −80 °C until processed.

### Bulk-seq library preparation

Samples were defrosted and washed with AMPure XP bead (BECKMAN COULTER, A63881) at 1:1 ratio. RNA libraries from the bulk tissues were prepared using mcSCRBseq protocol^84^ with minor modifications. RT reaction was applied directly on the beads with a final volume of 20 µl. RNase free water (8.4 μl) was added to the beads and mixed with 9.6 μl reaction buffer (1× Maxima H Buffer, 1 mM dNTPs, 2 μM TSO* E5V6NEXT, 7.5% PEG8000, 20U Maxima H enzyme, 1 μl barcoded RT primer). Subsequent steps were applied as mentioned in the protocol. Library final concentration of 2.4 pM was loaded on NextSeq 550 (Illumina) sequencing machine aiming for 20 M reads per sample. Raw files were converted to FASTQ files using bcl2fastq 2.17 (Illumina), to obtain the UMI counts, fastq reads were aligned to the mouse reference genome GRCm38.94 (Ensembl) using zUMI package^85^ with the following parameters: RD1 16 bp, RD2 66 bp with a barcode (i7) length of 8 bp. UMI counts for “coding_genes” were processed with the edgeR package running on R 3.5.1. Samples with less than 10k UMI counts were removed. Genes that were expressed in cpm >10 and in two or more samples were retained for the downstream analysis.

### Data processing

The final cpm (counts per million) table included 14 mice (*n* = 8, WT: (*n* = 3, PBS; *n* = 5, LPS); *n* = 6, HIF-1α^−/−^ (*n* = 3 per group)). We normalized the cpm values by the sum of all genes for each sample. We next divided the gene expression values in each sample by the sum for all genes that individually make up less than 1% of the sample’s summed expression values. For each analyzed group, we computed the means and standard errors of the means over the different mice. Only genes with mean values higher than 5 × 10^−6^ were considered.

### Bacterial preparation

*Salmonella typhimurium* (strain SL1344) were kindly provided by Roi Avraham (Weizmann Institute, Israel). *Salmonella* working concentration of 2 × 10^8^ cell/ml was prepared from an overnight inoculum in LB (Lysogeny broth) incubated at 37 °C with shaking. The bacteria were washed and diluted with PBS to the desired concentration in 200 µl and was administered i.p. at 7 A.M. All the mice were sacrificed at 11 A.M. PBS alone served as vehicle control. This *Salmonella* dose is nonlethal in strains applied for this study.

### l-Lactate measurement

The l-lactate assay was performed by using the l-lactate kit (700510, Cayman) which provides a fluorescence method according to the manufacturer’s instructions.

For detecting BM l-lactate, six bones from each mouse were flushed with 1 ml PBS and BM extracellular fluid samples (supernatants) were prepared according to the manufacturer’s instructions. BM supernatants of WT, NOX mutated (gp91phox^−/−^), and HIF-1α mutated (heterozygous and homozygous) mice were collected 4 h post LPS, and BM supernatants of WT mice collected 4 h post lactate or *Salmonella* treatments. For detecting blood l-lactate, plasma was collected from WT mice following LPS, lactate or Salmonella treatments and prepared according to the manufacturer’s instructions. The levels of BM lactate of WT mice treated with PBS varied between independent experiments (Figs. 1f, 7a, and Supplementary Fig. 2a), since new l-lactate kits were used in each experiment. However, the trend following LPS or *Salmonella* treatment was similar in all the experiments.

In this assay, lactate dehydrogenase catalyzes the oxidation of lactate to pyruvate, along with the concomitant reduction of NAD^+^ to NADH. NADH reacts with the fluorescence substrate to yield a highly fluorescent product, which is analyzed with an excitation wavelength of 530–540 nm and an emission wavelength of 585–595 nm.

### Imaging flow cytometry analysis

BM cells were prepared as indicated for flow cytometry, stained for extracellular markers (MCT1, MCT1, and GPR81) and analyzed using an ImageStreamX (Amnis corp., part of EMD-Millipore, Seattle, WA) machine. Samples were visualized and analyzed for the expression of markers with IDEAS 6.2 software (Amnis). Single stained control cells were used to compensate fluorescence between channel images to avoid emission spectra overlap. Cells were gated for single cells with the area and aspect ratio features and for focused cells, using the Gradient RMS feature. Cells were then gated for the selection of positively stained cells based on their pixel intensity, as set by the cutoff with secondary antibody control staining. Three samples from three mice were analyzed to confirm biological repeats of observed data.

### Establishment of chimeric mice

Recipient GPR81^−/−^, gp91phox^−/−^, Sca-1 eGFP or WT mice were subjected to irradiation (950 cGY) 16–20 h before transplantation. Irradiated mice received 8–10 million donor total BM cells (filtered and resuspended in RPMI^−/−^) and the cells were allowed to repopulate for 4 weeks before conducting experiments. Four weeks after transplantation, recipient (transplanted) mice were treated with lactate or LPS and sacrificed 4 h later at 11 A.M. to determine BM vascular permeability, neutrophil mobilization and metabolic changes in BM neutrophils. For neutrophil imaging, recipient Sca-1 eGFP mice were irradiated (as mentioned above) and received 8-10 million donor total BM cells (Catchup; Ly6G tdTomato). Four weeks after transplantation, chimeric mice were treated with PBS or lactate to track live neutrophil mobilization over time. Sca-1–eGFP transgenic mice were used to distinguish between Sca-1^low^ sinusoidal BMECs (sBMECs) from Sca-1^+^ arterial BMECs (aBMECs)^24^.

### Intravital imaging by two-photon laser scanning microscopy

Chimeric mice were anesthetized with a mixture of 50 mg ketamine, 15 mg xylazine and 2.5 mg acepromazine per kg of body weight at the indicated time points after lactate or PBS administration. Mice were mounted in a custom-designed heated mouse holder and the skin above the calvaria was cut to reveal the underlying calvarial bone. The mouse was immobilized to the heated holder, a drop of PBS was applied to the skull, covered with a glass coverslip and placed under the microscope water-dipping objective (Zeiss 20 × 1.05 NA plan objective). Zeiss LSM 880 upright microscope fitted with Coherent Chameleon Vision laser was used. Images were acquired with a femtosecond-pulsed two-photon laser tuned to 940 nm. The microscope was fitted with a filter cube containing 565 LPXR to split the emission to a PMT detector (with a 579–631 nm filter for tdTomato fluorescence) and to an additional 505 LPXR mirror to further split the emission to two GaAsp detectors (with a 500–550 nm filter for GFP fluorescence). For in vivo intravital imaging experiments, images were acquired as 70–100 µm z-stacks with 3–5 µm steps between each z-plane (15–19 frames) at 30 s interval for 30–50 min. Static images were acquired as 30–100 µm z-stacks with 1–3 μm steps between each z-plane (20–50 frames). The zoom was set to 0.7, and images were acquired at 512 × 512 *x*–*y* resolution. Static images were acquired following mice scarification 4 h post treatment. Sca-1 eGFP recipient mice reconstituted with Catchup donor BM (Ly6G tdTomato) cells were used to endogenously label blood vessels (Sca-1 marker distinguish between Sca-1^low^ sinusoidal BMECs (sBMECs) from Sca-1^+^ arterial BMECs (aBMECs)^24^) and neutrophils, respectively throughout all intravital imaging experiments. The second harmonic generation (SHG) signal was used to detect the inner bone surface (endosteum) and detect the BM cavities.

### Quantification of the total volume of neutrophils inside the sinus

Quantification of the total volume of neutrophils inside the sinus area over time was done using Imaris software [v9.5.1, Bitplane AG], as followed: Neutrophils express Ly6G tdTomato, while the sinusoidal- and arterial endothelial cells express Sac-1 eGFP with different intensity (sinusoid: Sac-1^low^; arterials: Sca-1^high^^24^). Sca-1 eGFP signal is very bright and bleeds through into the tdTomato channel. To enable detection of neutrophils only, first a new channel was generated by subtracting the Sca-1 eGFP channel from the Ly6G tdTomato channel and then manually masking the new channel in the sinus area using Imaris manual surface option. Imaris Surface was used to segment neutrophils inside the sinus automatically (using local contrast option, fix threshold, and discarding very small objects) and then measured their total volume for all time points.

### In vivo EBD penetration to the BM

BM vascular endothelial barrier function was assessed using the EBD assay. Evans Blue (Sigma) dissolved with PBS was filtered and injected i.v. to mice. For the lactate, GPR81 agonist, Epac1 agonist, and LPS treated mice; 20 mg/ml EBD was injected 4 h before mice were sacrificed. For neutrophil depletion, the anti-Ly6G antibody was administered i.p. at 24 h prior to EBD and LPS injections. In each experiment, a non-injected mouse was used for control blank measurements. Subsequently, mice were perfused with PBS via the left ventricle to remove the intravascular dye. Femurs were removed and formamide was used for bone flushing, crushing and chopping. EBD was extracted in formamide by incubation and shaking of flushed and crushed fractions, overnight at 60 °C. After 30 min centrifugation at 2000*g*, EBD in BM supernatants was quantitated by spectrophotometric analysis at 620 nm.

### Immunofluorescence

Femurs fixed overnight at 4 °C in 4% paraformaldehyde followed by 30% sucrose exchange for 2 days, were embedded in optimal cutting temperature compound (Sakura Finetek USA, Inc., Tissue-Tek) and ‘snap-frozen’ in N-methylbutane chilled in liquid nitrogen. Sections of 8μm thickness were generated with a cryostat (Leica) at −24 °C with a tungsten carbide blade (Leica) and a CryoJane tape transfer system (Instrumedics). Sections were mounted on adhesive-coated slides (Leica), fixed in acetone and air-dried. Sections were permeabilized with 0.1% Triton for 8 min, blocked in 20% normal horse serum (Vector) and stained overnight at 4 °C with primary antibodies. Then sections were incubated for 1 h with secondary antibodies and stained for 5 min in RT with Hoechst33342 (Molecular Probes). Endomucin was stained using rat anti-mouse Endomucin (1:50, clone V.7C7, Santa Cruz), followed by donkey anti-rat Alexa 488 (1:400, Jackson ImmunoResearch). Femoral sections from GPR81-RFP and WT mice were stained with rabbit anti-mouse RFP (1:100, MBL), followed by donkey anti-rabbit Rhodamin Red X conjugated antibody (1:200, Jackson ImmunoResearch).

Confocal imaging was performed using an upright Leica TCS SP8, equipped with internal Hybrid (HyD) detectors and Acusto Optical Tunable Filter (Leica microsystems CMS GmbH, Germany). Excitation of Rhodamin Red X was done at a wavelength of 561 nm by DPSS laser. Excitation of Alexa488 was done at a wavelength of 488 nm by Argon laser. Excitation of Hoechst33342 was done by Diode405 laser. Emission signal for Rhodamin Red X, Alexa488 and Hoechst was collected using the HyD detectors at the range of 588–640, 497–555, and 413–501 nm, respectively. Images were acquired using the 8 k Hz resonant/galvometric scanner in a 1024 × 1024 format using 20X air objective (HC PL APO 20×/0.75 CS2) and application zoom factor of 7 or 63× oil immersion objective (HC PL APO 63×/1.40 OIL CS2). Z stack acquisition was performed using 14 z steps while only one slice was selected for presentation. The presented slice has pixel width 0.077 µm, pixel height 0.077 µm, and voxel depth 0.999 µm. The acquired images were processed using ImageJ software to enhance the contrast.

### Chemokine quantification in BM and plasma

CXCL1 and CXCL2 protein amount were measured in BM samples. CXCL1, CXCL2, and G-CSF were measured in plasma samples taken 4 h post lactate injection using commercial ELISA reagents, following the manufacturer’s protocol (R&D Systems). Total protein content in BM fluids was quantified by Bradford assay. CXCL1/2 concentration in the BM fluids was normalized per protein content.

### Statistical analyses

All statistical analyses were conducted with Prism 8.0c version (^*^p < 0.05, ^**^p < 0.01, ^***^p < 0.001, ^****^p < 0.0001, n.s. represents nonsignificant). All data are expressed and presented as mean ± standard error of the mean (s.e.m.) and all *n* numbers represent biological repeats. Unless indicated otherwise in figure legends, a Student’s two-tailed unpaired *t* test was used to determine the significance of the difference between means of two groups. One-way ANOVA or two-way ANOVA was used to compare means among three or more independent groups. Tukey or Bonferroni post hoc tests were used to compare all pairs of treatment groups when the overall *P* value was <0.05. All mutated or transgenic mice were assigned according to their genotype. Litter mates and sex-matched animals were used whenever possible. All other parameters are random. The authors were not blinded to allocation during experiments.

### Reporting summary

Further information on research design is available in the Nature Research Reporting Summary linked to this article.

## Supplementary information

Supplementary Information

Description of Additional Supplementary Files

Supplementary Movie 1

Supplementary Movie 2

Reporting Summary

## Data Availability

Data have been deposited in the GenBank (Gene Expression Omnibus; GEO) with the accession code GSE143978. All other data are available within the manuscript and Supplementary Information files and raw data are included as a Source Data file.

## Source data

Source Data
