# Supplementary material for: Lactate released by inflammatory bone marrow neutrophils induces their mobilization via endothelial GPR81 signaling
Source: Nat Commun. 2020 Jul 15;11:3547. doi: 10.1038/s41467-020-17402-2 (PMC7363928; doi:10.1038/s41467-020-17402-2)
Supplement: Supplementary file 1 — Supplementary Information [file 41467_2020_17402_MOESM1_ESM.pdf]

Supplementary information

# Lactate released by inflammatory bone marrow neutrophils induces their mobilization via endothelial GPR81 signaling.

Khatib-Massalha et al.

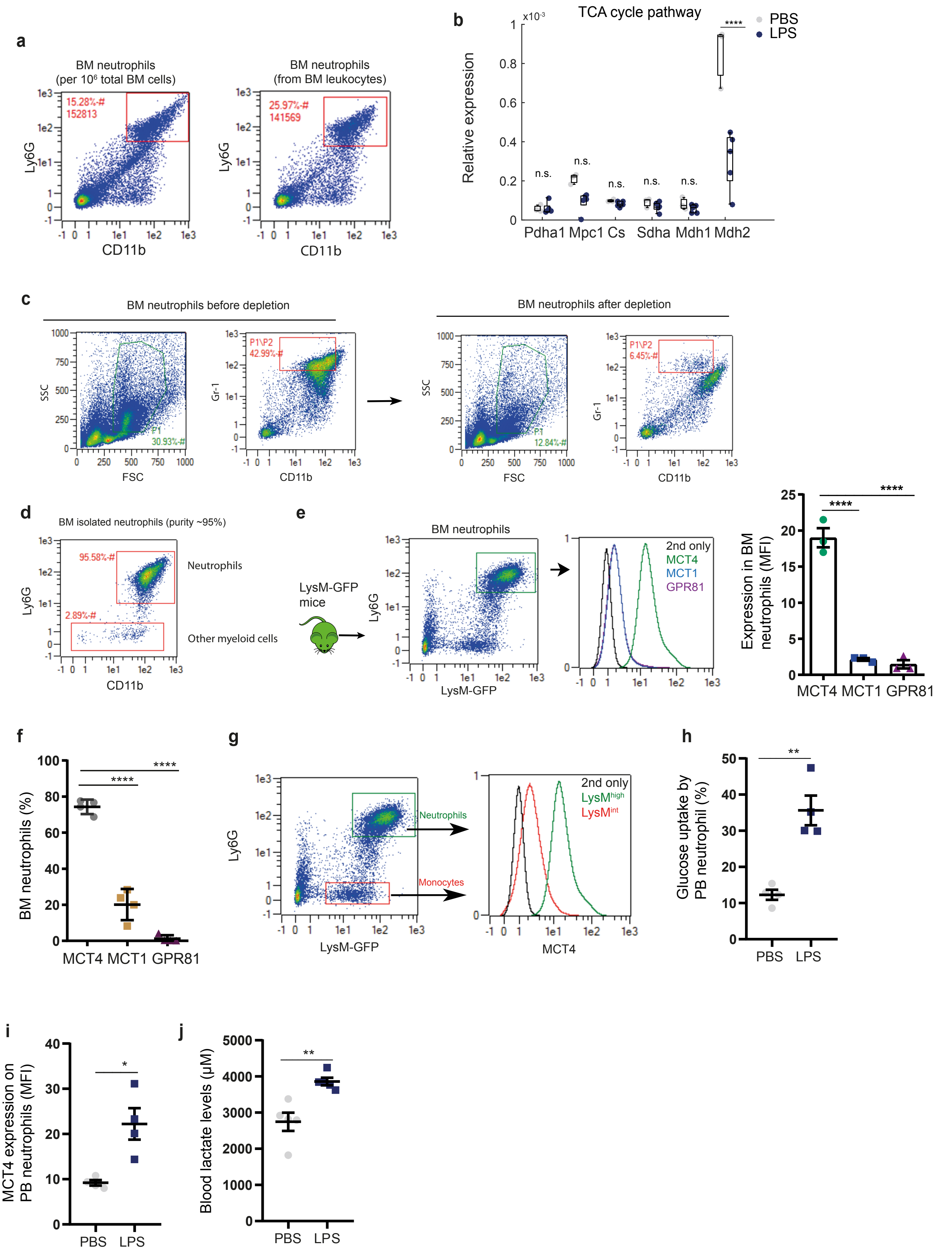
a
BM neutrophils
(per 10⁶ total BM cells)
15.28%-#
152813
Ly6G
CD11b
BM neutrophils
(from BM leukocytes)
25.97%-#
141569
Ly6G
CD11b
b
TCA cycle pathway
x10-3
Relative expression
PBS
LPS
n.s.
****
Pdha1 Mpc1 Cs Sdha Mdh1 Mdh2
c
BM neutrophils before depletion
SSC
FSC
P1
30.93%-#
Gr-1
CD11b
P1\P2
42.99%-#
BM neutrophils after depletion
SSC
FSC
P1
12.84%-#
Gr-1
CD11b
P1\P2
6.45%-#
d
BM isolated neutrophils (purity ~95%)
95.58%-#
Neutrophils
2.89%-#
Other myeloid cells
Ly6G
CD11b
e
LysM-GFP
mice
BM neutrophils
Ly6G
LysM-GFP
2nd only
MCT4
MCT1
GPR81
Expression in BM neutrophils (MFI)
****
MCT4 MCT1 GPR81
f
BM neutrophils (%)
****
MCT4 MCT1 GPR81
g
Ly6G
LysM-GFP
Neutrophils
Monocytes
2nd only
LysMhigh
LysMint
MCT4
h
Glucose uptake by PB neutrophil (%)
**
PBS LPS
i
MCT4 expression on PB neutrophils (MFI)
*
PBS LPS
j
Blood lactate levels (µM)
**
PBS LPS

**Supplementary Figure 1 related to Figure 1: BM neutrophils are highly glycolytic cells with low rate of oxidative phosphorylation. a,** Representative flow cytometry density plots to determine neutrophils ($CD11b^{high}/Ly6G^{high}$) frequency in total $10^6$ acquired BM cells (left panel) or to determine neutrophils frequency among BM leukocytes. **b,** TCA cycle enzymatic gene expression in sorted BM neutrophils following LPS treatment (WT; n=3, PBS; n=5, LPS). On each box, the bottom, middle and the top edges indicate the $25^{th}$, $50^{th}$ and $75^{th}$ percentiles, respectively. The whiskers extend to the most extreme data points. **c,** Representative flow cytometry density plots for BM neutrophils ($CD11b^{high}/Gr\text{-}1^{high}$) before and after neutrophils depletion. **d,** A representative flow cytometry density plot for BM neutrophils following neutrophils isolation. **e,** A representative flow cytometry density plot for BM neutrophils in steady state using LysM-GFP reporter mice, and a representative histogram plot and quantitative analysis of MCT4, MCT1 and GPR81 expression (n=3). **f,** Percentage of $MCT4^+$, $MCT1^+$ and $GPR81^+$ neutrophils in BM from WT mice measured and visualized by ImageStream analysis (n=4). **g,** A representative flow cytometry density plot of BM neutrophils (green; $LysM^{high}/Ly6G^{high}$) and monocytes (red; $LysM^{int}/Ly6G^{neg}$) in steady state using LysM-GFP reporter mice and a representative histogram plot showing MCT4 expression (n=3). **h-i,** Quantitative analysis of (**h)** 2-NBDG-glucose uptake by PB neutrophils (n=4; **p=0.0017) or (**i**) MCT4 expression on PB neutrophils (n=4; *p=0.0103) 4hr following LPS treatment in WT mice**. j,** Blood (plasma) lactate levels in WT mice treated with PBS or LPS (n=5); **p=0.0036. Data are represented as mean ± SEM from 2-4 independent experiments. *P<0.05; **P<0.01; ****P<0.0001, Two-way ANOVA with Bonferroni *post hoc* test (b), one-way ANOVA with Tukey's *post hoc* test (e and f) or student's two-tailed unpaired t-test (h-j).

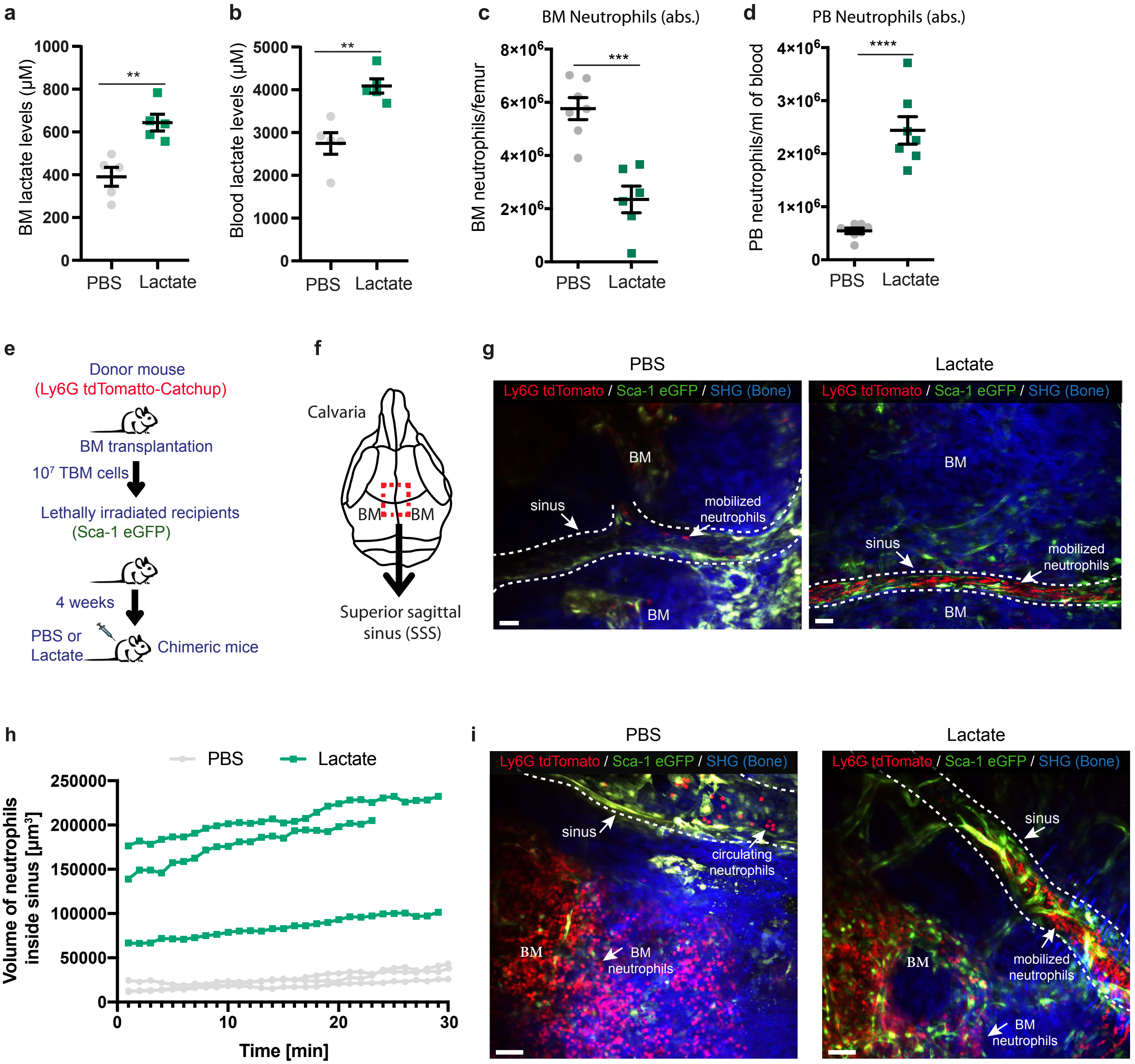
a
BM lactate levels (μM)
PBS
Lactate
**
b
Blood lactate levels (μM)
PBS
Lactate
**
c
BM Neutrophils (abs.)
BM neutrophils/femur
***
d
PB Neutrophils (abs.)
PB neutrophils/ml of blood
****
e
Donor mouse
(Ly6G tdTomatto-Catchup)
BM transplantation
10^7 TBM cells
Lethally irradiated recipients
(Sca-1 eGFP)
4 weeks
PBS or
Lactate
Chimeric mice
f
Calvaria
BM
BM
Superior sagittal
sinus (SSS)
g
PBS
Lactate
Ly6G tdTomato / Sca-1 eGFP / SHG (Bone)
BM
sinus
mobilized
neutrophils
h
PBS
Lactate
Volume of neutrophils
inside sinus [μm³]
Time [min]
i
PBS
Lactate
Ly6G tdTomato / Sca-1 eGFP / SHG (Bone)
sinus
circulating
neutrophils
BM
BM
neutrophils
mobilized
neutrophils

**Supplementary Figure 2 related to Figure 2: Lactate preferentially induces rapid BM neutrophil mobilization. a-b,** Lactate levels in (a) BM or (b) blood from WT mice treated with PBS or lactate (n=5); (a)**p=0.0026; (b)**p=0.0022. **c,** Absolute numbers of BM neutrophils per femur 4hr following 50.5 mg lactate injection (n=7, PBS; n=6, lactate; ***p=0.003). **d,** Absolute numbers of circulating neutrophils per ml of blood (n =7). e, A schematic illustration of chimeric mice generated by transplantation of total BM cells from Catchup (Ly6G tdTomato) donors into Sca-1 eGFP recipient mice, treated with lactate. **f,** A schematic illustration of the calvaria structure. Red dashed box indicates superior sagittal sinus (SSS) between two sides of BM. **g,** Representative TPLSM images showing neutrophils inside the sinus from chimeric mice ~3hr following PBS (left panel) or lactate (right panel) administration. Images were taken from intravital imaging videos. Scale bar, 50 μm. The videos are as available in the Supplementary Material. **h,** Quantification of neutrophil volumes inside the sinus (SSS) from videos acquired by TPLSM (~3hr post treatment; n=3 per group), within the calvarial bone. **i,** Representative 3D TPLSM images 4hr post PBS (left panel) or lactate (right panel) treatment, in the calvarial bone of chimeric mice as in (e). Ly6G tdTomato marked the neutrophils (red), Sca-1 eGFP marked the endothelial cells (green) SHG marked the bone (blue). White dashed lines indicate superior sagittal sinus (calvaria central sinus), arrows indicate BM, circulating or mobilized neutrophils. Representative images out of 3 independent experiments are shown. Scale bar, 50 μm. Data are represented as mean ± SEM from 3-5 independent experiments. **P<0.01; ***P<0.001; ****P<0.0001, Student's two-tailed unpaired t-test (a-d).

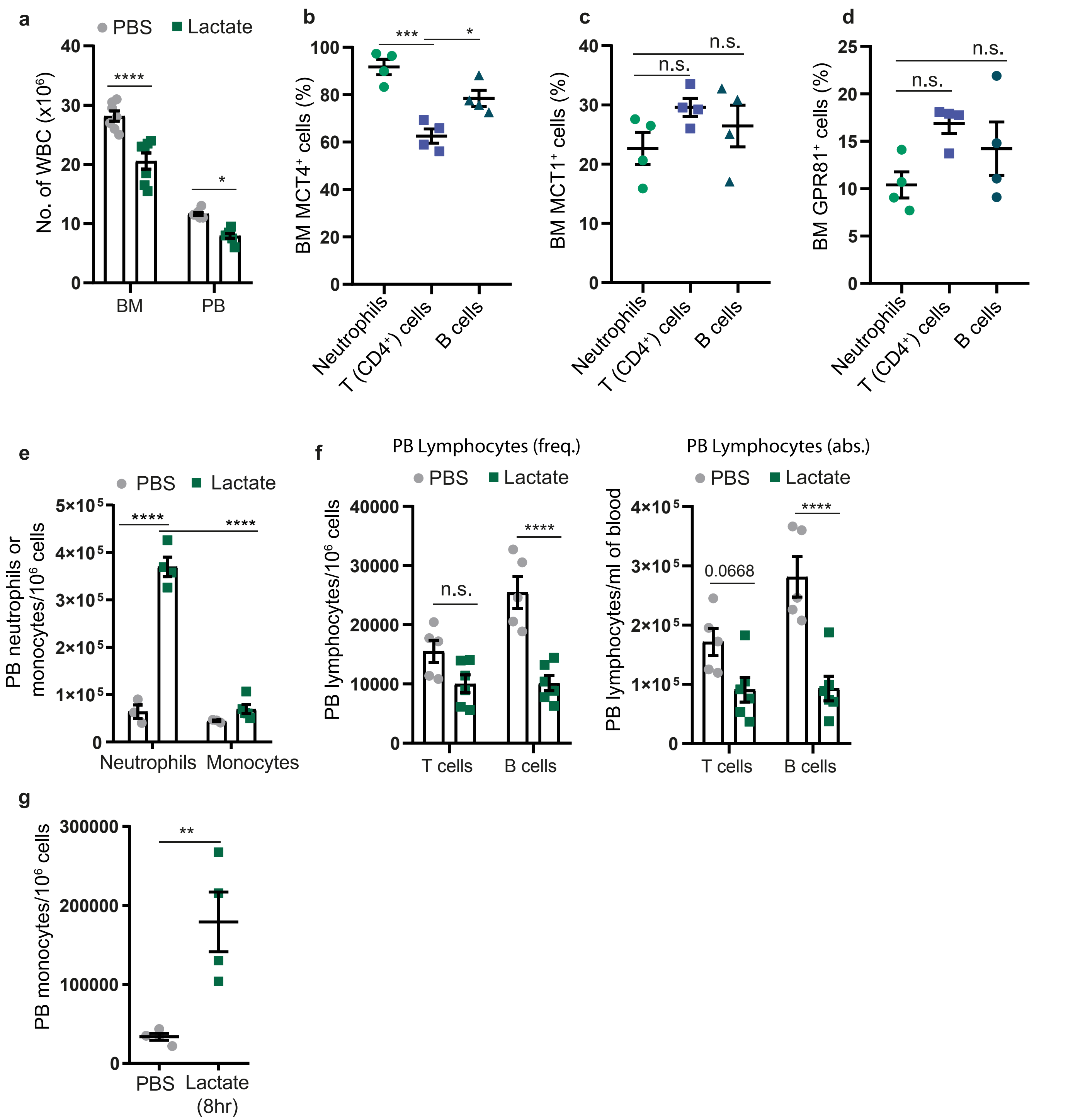
a
PBS
Lactate
No. of WBC (x10^6)
0
10
20
30
40
****
*
BM
PB
b
BM MCT4+ cells (%)
0
20
40
60
80
100
***
*
Neutrophils
T (CD4+) cells
B cells
c
BM MCT1+ cells (%)
0
10
20
30
40
n.s.
n.s.
Neutrophils
T (CD4+) cells
B cells
d
BM GPR81+ cells (%)
0
5
10
15
20
25
n.s.
n.s.
Neutrophils
T (CD4+) cells
B cells
e
PBS
Lactate
PB neutrophils or monocytes/10^6 cells
0
1×10^5
2×10^5
3×10^5
4×10^5
5×10^5
****
****
Neutrophils
Monocytes
f
PB Lymphocytes (freq.)
PBS
Lactate
PB lymphocytes/10^6 cells
0
10000
20000
30000
40000
n.s.
****
T cells
B cells
PB Lymphocytes (abs.)
PBS
Lactate
PB lymphocytes/ml of blood
0
1×10^5
2×10^5
3×10^5
4×10^5
0.0668
****
T cells
B cells
g
PB monocytes/10^6 cells
0
100000
200000
300000
**
PBS
Lactate (8hr)

**Supplementary Figure 3 related to Figure 2: Lactate preferentially induces neutrophil mobilization rather than other BM cells subsets. a,** Number of BM WBC per femur and PB WBC per ml of blood 4hr following lactate treatment (n=7; *p=0.0229). **b-d,** Percentage of (b) $MCT4^+$, (c) $MCT1^+$ and (d) $GPR81^+$ BM neutrophils, CD4 T cells and B cells from WT mice during steady state (n=4; (b)***p=0.0003; *p=0.0170). **e,** Frequency of neutrophils (n=3, PBS; n=4, 50.5 mg lactate) and monocytes (n=3, PBS; n=5, 50.5 mg lactate) in PB from LysM-GFP reporter mice 4hr following lactate treatment. **f,** Frequency and absolute numbers of PB lymphocytes (n=5, PBS; n=6, lactate) following 4hr lactate injection. **g,** Monocyte ($CD11b^{intm}$/$Ly6C^{high}$) frequency in PB 8hr following treatment with 50.5 mg lactate in WT mice (n=4; **p=0.0087). Data are represented as mean ± SEM from 2-3 independent experiments. *P<0.05; **P<0.01; ***P<0.001; ****P<0.0001, Student's two-tailed unpaired t-test (g), one-way ANOVA with Tukey's *post hoc* test (b-d) or two-way ANOVA with Tukey's *post hoc* test (a, e-f).

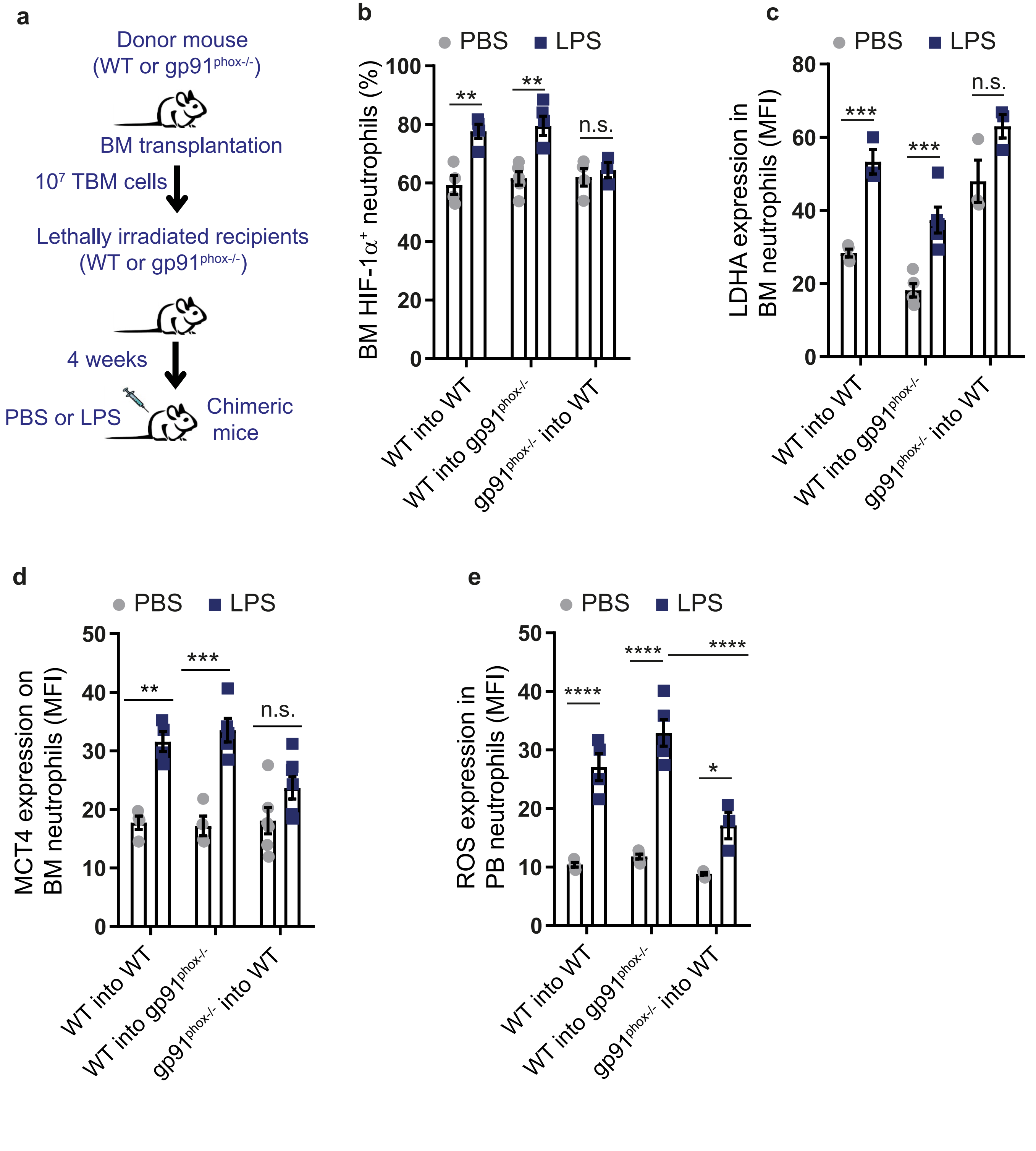
a
Donor mouse
(WT or gp91phox-/-)
BM transplantation
10^7 TBM cells
Lethally irradiated recipients
(WT or gp91phox-/-)
4 weeks
PBS or LPS
Chimeric mice
b
PBS LPS
BM HIF-1α+ neutrophils (%)
**
**
n.s.
WT into WT
WT into gp91phox-/-
gp91phox-/- into WT
c
PBS LPS
LDHA expression in BM neutrophils (MFI)
***
***
n.s.
WT into WT
WT into gp91phox-/-
gp91phox-/- into WT
d
PBS LPS
MCT4 expression on BM neutrophils (MFI)
**
***
n.s.
WT into WT
WT into gp91phox-/-
gp91phox-/- into WT
e
PBS LPS
ROS expression in PB neutrophils (MFI)
****
****
****
*
WT into WT
WT into gp91phox-/-
gp91phox-/- into WT

**Supplementary Figure 4 related to Figure 3: NOX activity in BM neutrophils is essential for lactate production and release**. **a,** A schematic illustration of chimeric mice generated by transplantation of total BM cells from gp91phox$^{-/-}$ or WT donors into WT or gp91phox$^{-/-}$ recipient mice, treated with LPS. **b,** Percentage of HIF-1α$^{+}$ neutrophils in the BM of chimeric mice (n=4, WT into WT +PBS or LPS; n=5, WT into gp91phox$^{-/-}$ +PBS or LPS; n=4, gp91phox$^{-/-}$ into WT+PBS; n=3, gp91phox$^{-/-}$ into WT+LPS). **c,** Quantitative analysis of LDHA expression in BM neutrophils (n=4, WT into WT +PBS; n=3, WT into WT+LPS; n=5, WT into gp91phox$^{-/-}$ +PBS or LPS; n=3, gp91phox$^{-/-}$ into WT+PBS or LPS). **d,** Quantitative analysis of surface MCT4 expression on BM neutrophils (n=4, WT into WT +PBS or LPS; n=4, WT into gp91phox$^{-/-}$ +PBS; n=5, WT into gp91phox$^{-/-}$ +LPS; n=6, gp91phox$^{-/-}$ into WT+PBS; n=7, gp91phox$^{-/-}$ into WT+LPS). **e,** Quantitative analysis of ROS production in PB neutrophils (n=4, WT into WT+PBS or LPS; n=5, WT into gp91phox$^{-/-}$ +PBS or LPS; n=4, gp91phox$^{-/-}$ into WT+PBS; n=3, gp91phox$^{-/-}$ into WT+LPS). Data are represented as mean ± SEM from 2 independent experiments. *P<0.05; **P<0.01; ***P<0.001; ****P<0.0001, Two-way ANOVA with Tukey's *post hoc* test (b-e).

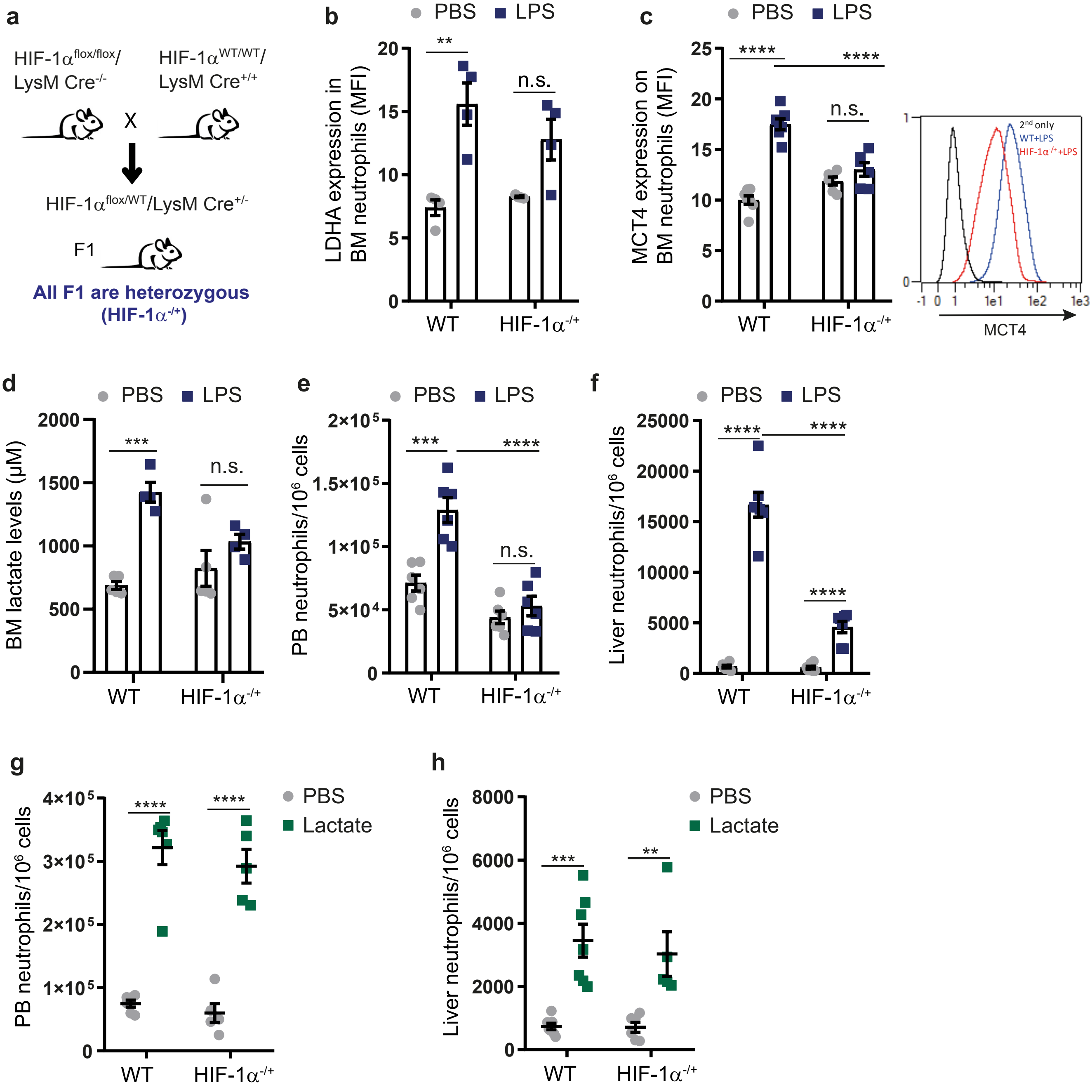
a
HIF-1α flox/flox/ LysM Cre-/-
HIF-1α WT/WT/ LysM Cre+/+
X
HIF-1α flox/WT/LysM Cre+/-
F1
All F1 are heterozygous (HIF-1α-/+)
b
PBS
LPS
LDHA expression in BM neutrophils (MFI)
**
n.s.
WT
HIF-1α-/+
c
MCT4 expression on BM neutrophils (MFI)
****
****
n.s.
2nd only
WT+LPS
HIF-1α-/++LPS
MCT4
d
BM lactate levels (μM)
***
n.s.
e
PB neutrophils/10⁶ cells
***
****
n.s.
f
Liver neutrophils/10⁶ cells
****
****
****
g
PB neutrophils/10⁶ cells
****
****
Lactate
h
Liver neutrophils/10⁶ cells
***
**

**Supplementary Figure 5 related to Figure 4**: **HIF-1α mediates lactate release by inflammatory neutrophils and their mobilization**. **a,** A schematic illustration of the generation of specific myeloid-selective HIF-1α deficiency in mice using LysM Cre and the HIF-1α gene flanked by LoxP. **b,** Quantitative analysis of LDHA expression in BM neutrophils from WT vs. HIF-1α$^{-/+}$ following either PBS or LPS treatment (n=4; **p=0.0025). **c,** Quantitative analysis and a representative flow cytometry histogram plot of MCT4 expression on BM neutrophils following LPS treatment (n=7, WT; n=6, HIF-1α$^{-/+}$). **d,** BM lactate levels in WT (n=5, PBS; n=4, LPS; ***p=0.0003) vs. HIF-1α$^{-/+}$(n=5, PBS; n=4, LPS) mice. **e,f,** Frequency of PB (e, n=6) and liver (f, n=7) neutrophils after LPS injection in WT vs. HIF-1α$^{-/+}$ mice. **g,** PB neutrophil frequency in WT (n=6) vs. HIF-1α$^{-/+}$ (n=5) mice following lactate treatment. **h,** Liver neutrophil frequency in WT (n=7; ***p=0.0004) and HIF-1α$^{-/+}$ mice (n=6, PBS; n=5, lactate; **p=0.0068) treated with lactate. Data are represented as mean ± SEM from 3-4 independent experiments. *P<0.05; **P<0.01; ***P<0.001; ****P<0.0001, Two-way ANOVA with Tukey's post hoc test (b-h).

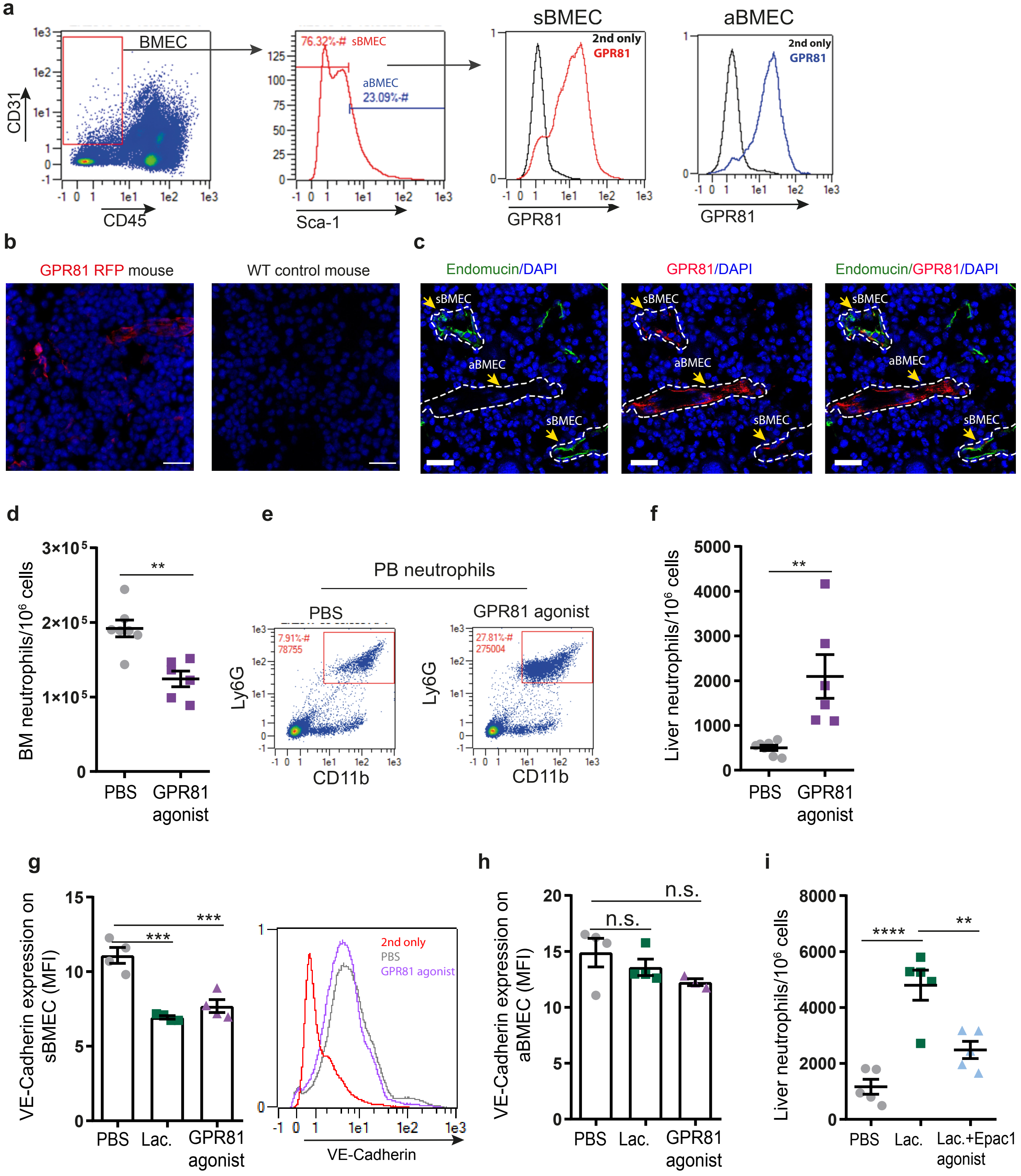

a
BMEC
CD31
CD45
76.32%-# sBMEC
aBMEC
23.09%-#
Sca-1
sBMEC
2nd only
GPR81
aBMEC
2nd only
GPR81
GPR81
b
GPR81 RFP mouse
WT control mouse
c
Endomucin/DAPI
GPR81/DAPI
Endomucin/GPR81/DAPI
sBMEC
aBMEC
d
BM neutrophils/10^6 cells
PBS
GPR81 agonist
e
PB neutrophils
PBS
GPR81 agonist
7.91%-# 78755
27.81%-# 275004
Ly6G
CD11b
f
Liver neutrophils/10^6 cells
PBS
GPR81 agonist
g
VE-Cadherin expression on sBMEC (MFI)
PBS
Lac.
GPR81 agonist
2nd only
PBS
GPR81 agonist
VE-Cadherin
h
VE-Cadherin expression on aBMEC (MFI)
n.s.
PBS
Lac.
GPR81 agonist
i
Liver neutrophils/10^6 cells
PBS
Lac.
Lac.+Epac1 agonist

**Supplementary Figure 6 related to Figure 5: Lactate-activated endothelial GPR81 signaling down-regulates surface VE-Cadherin expression. a,** Flow cytometry density plots of sinusoidal and arterial BM endothelial cells (s/a BMECs respectively) and representative histogram plots of GPR81 expression on both sBMEC and aBMEC in WT mice. The data are from one representative experiment out of 2 independent experiments. **b,** Representative fluorescent images from 3 independent experiments of GPR81 (red) and nuclei (blue; DAPI) in the femoral diaphysis of GRP81-RFP reporter and WT control mice. Scale bar indicates 20 μm. **c,** Representative fluorescent images from 3 independent experiments of Endomucin (green), GPR81 (red), and nuclei (blue; DAPI) in the femoral diaphysis of GRP81-RFP reporter mice; arrows indicate Endomucin$^{-}$/GPR81$^{+}$ aBMEC and Endomucin$^{+}$/GPR81$^{+}$ sBMEC. Scale bar indicates 20 μm. **d,** Frequency of BM neutrophils 4hr following GPR81 agonist treatment (n=7, PBS; n=6, GPR81 agonist; **p=0.0012). **e,** Representative flow cytometry density plots of PB neutrophils following PBS vs. GPR81 agonist treatment. **f,** Neutrophil frequency following GPR81 agonist treatment in liver (n=7, PBS; n=6, GPR81 agonist; **p=0.0049). **g,** Quantitative analysis and representative flow cytometry histogram plot of surface VE-Cadherin expression on sBMEC 30min post lactate or GPR81 agonist injection (n=4 per group). **h,** Surface VE-Cadherin expression on aBMEC 30min post injection (n=4, PBS or lactate; n=3, GPR81 agonist). **i,** Frequency of neutrophils in liver following PBS, lactate or lactate with Epac1 agonist (n=5 per group; **p(lactate vs. lactate+Epac1 agonist)=0.0033). Data are represented as mean ± SEM from 3-5 independent experiments. **P<0.01; ***P<0.001; ****P<0.0001, Student's two-tailed unpaired t-test (d and f) or one-way ANOVA with Tukey's post hoc test (g-i).

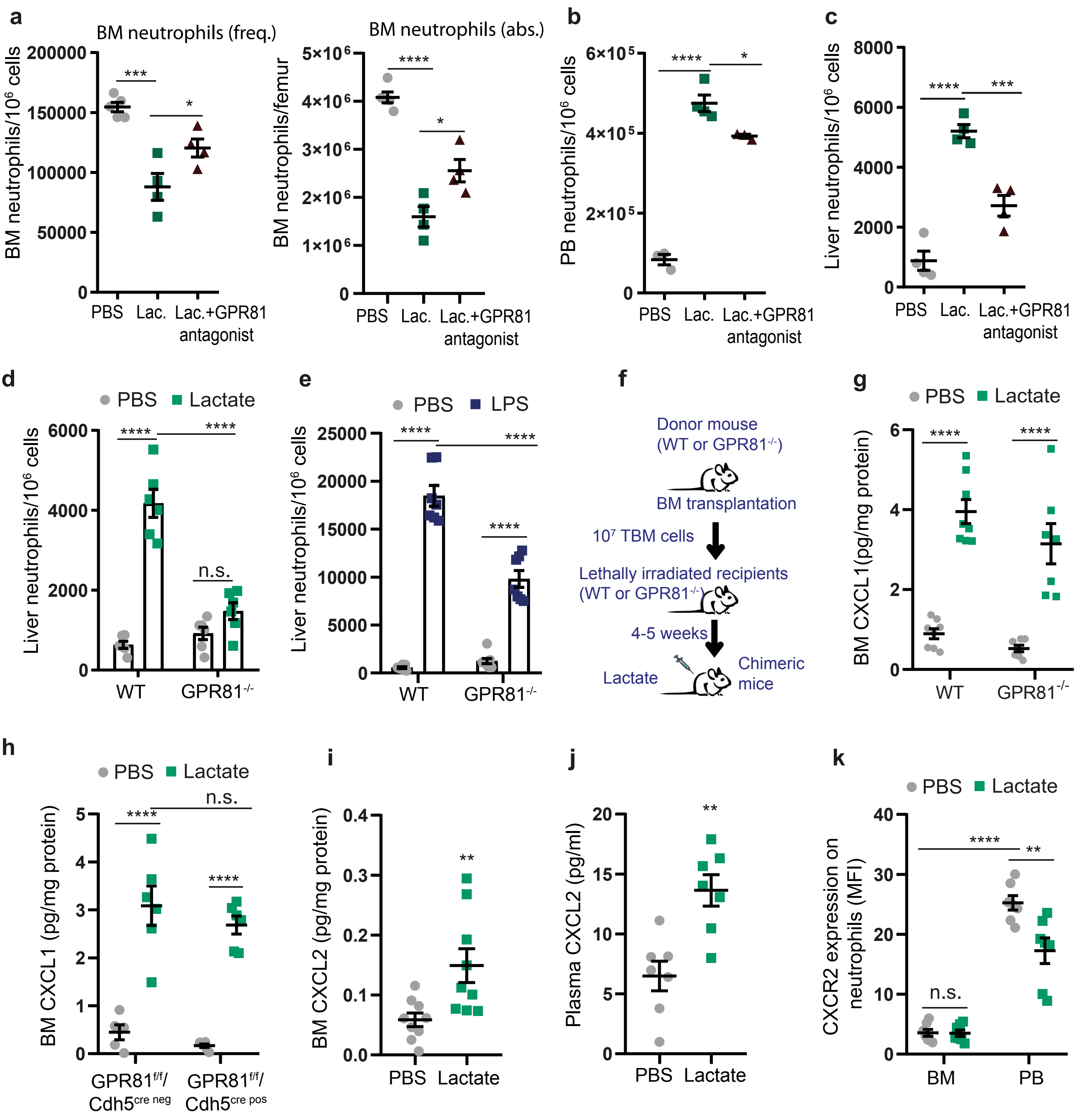
a
BM neutrophils (freq.)
BM neutrophils/10^6 cells
PBS
Lac.
Lac.+GPR81 antagonist
BM neutrophils (abs.)
BM neutrophils/femur
b
PB neutrophils/10^6 cells
c
Liver neutrophils/10^6 cells
d
PBS
Lactate
n.s.
WT
GPR81-/-
e
LPS
f
Donor mouse (WT or GPR81-/-)
BM transplantation
10^7 TBM cells
Lethally irradiated recipients (WT or GPR81-/-)
4-5 weeks
Lactate
Chimeric mice
g
BM CXCL1(pg/mg protein)
h
BM CXCL1 (pg/mg protein)
GPR81f/f/ Cdh5cre neg
GPR81f/f/ Cdh5cre pos
i
BM CXCL2 (pg/mg protein)
j
Plasma CXCL2 (pg/ml)
k
CXCR2 expression on neutrophils (MFI)
BM
PB

**Supplementary Figure 7 related to Figure 6: Lactate-induced neutrophil mobilization is mediated by GPR81-dependent and independent mechanisms. a-c,** Frequency of neutrophils in BM (a, n=5, PBS; n=4, lactate or lactate + GPR81 antagonist-3-OBA; ***p(PBS vs. lactate)=0.0002; *p(lactate vs. lactate+3-OBA)=0.038), in PB (b, n=3, PBS; n=4, lactate; n=3, lactate + GPR81 antagonist), or in liver (c, n=4 per group). **d,** Frequency of liver neutrophils following lactate treatment in WT vs. GPR81$^{-/-}$ mice (n=6). **e,** Frequency of liver neutrophils following LPS treatment in WT vs. GPR81$^{-/-}$ mice (WT; n=8, PBS; n=7, LPS; GPR81$^{-/-}$; n=8, PBS; n=7, LPS). **f,** A schematic illustration of chimeric mice generated by transplantation of BM cells from GPR81$^{-/-}$ or WT donors into WT or GPR81$^{-/-}$ recipient mice, treated with lactate. **g,h,** BM protein levels of CXCL1 in **(g)** WT vs. GPR81$^{-/-}$ mice (WT; n=8; GPR81$^{-/-}$; n=7) or **(h)** GPR81$^{f/f}$/Cdh5$^{cre\ neg}$ (n=5, PBS; n=6, lactate) vs. GPR81$^{f/f}$/Cdh5$^{cre\ pos}$ mice (n=6) following lactate treatment. **i,** BM protein levels of CXCL2 (n=9; **p=0.0090) in WT mice following lactate administration. **j,** Plasma levels of CXCL2 (n=7; **p=0.0019) in WT mice following lactate administration. **k,** Quantitative analysis of CXCR2 expression on BM and PB neutrophils from WT mice treated with lactate (n=7, BM or PB; **p(PB+PBS vs. PB+lactate)=0.0011). Data are represented as mean ± SEM from 3-5 independent experiments. *P<0.05; **P<0.01; ***P<0.001; ****P<0.0001, Student's two-tailed unpaired t-test (i-j), one-way ANOVA with Tukey's *post hoc* test (a-c) or two-way ANOVA with Tukey's *post hoc* test (d-e, g-h, k).
