# Supplementary material for: Lactate released by inflammatory bone marrow neutrophils induces their mobilization via endothelial GPR81 signaling
Source: Nat Commun. 2020 Jul 15;11:3547. doi: 10.1038/s41467-020-17402-2 (PMC7363928; doi:10.1038/s41467-020-17402-2)
Supplement: Supplementary file 2 — Description of Additional Supplementary Files [file 41467_2020_17402_MOESM2_ESM.pdf]

Description of Additional Supplementary Files

Supplementary Video 1

Description: Time-lapse imaging of transplanted Ly6G tdTomato neutrophils (red) in Sca-1 eGFP (green) recipient mouse imaged by TPLSM, ~3hr following PBS treatment. Images were max projected over 70-100µm z-stacks. Scale bar, 100µm. Representative video of 3 independent experiments with one mouse in each experiment.

Supplementary Video 2

Description: Time-lapse imaging of transplanted Ly6G tdTomato neutrophils (red) in Sca-1 eGFP (green) recipient mouse imaged by TPLSM, ~3hr following lactate treatment. Images were max projected over 70-100µm z-stacks. Scale bar, 100µm. Representative video of 3 independent experiments with one mouse in each experiment.
