# Supplementary material for: Lactate released by inflammatory bone marrow neutrophils induces their mobilization via endothelial GPR81 signaling
Source: Nat Commun. 2020 Jul 15;11:3547. doi: 10.1038/s41467-020-17402-2 (PMC7363928; doi:10.1038/s41467-020-17402-2)
Supplement: Supplementary file 5 — Reporting Summary [file 41467_2020_17402_MOESM5_ESM.pdf]

nature research

Corresponding author(s): Tsvee Lapidot
Last updated by author(s): Jun 4, 2020

Reporting Summary

Nature Research wishes to improve the reproducibility of the work that we publish. This form provides structure for consistency and transparency in reporting. For further information on Nature Research policies, see our Editorial Policies and the Editorial Policy Checklist.

Statistics

For all statistical analyses, confirm that the following items are present in the figure legend, table legend, main text, or Methods section.

n/a	Confirmed	
☐	☒	The exact sample size (*n*) for each experimental group/condition, given as a discrete number and unit of measurement
☐	☒	A statement on whether measurements were taken from distinct samples or whether the same sample was measured repeatedly
☐	☒	The statistical test(s) used AND whether they are one- or two-sided<br>*Only common tests should be described solely by name; describe more complex techniques in the Methods section.*
☐	☒	A description of all covariates tested
☒	☐	A description of any assumptions or corrections, such as tests of normality and adjustment for multiple comparisons
☐	☒	A full description of the statistical parameters including central tendency (e.g. means) or other basic estimates (e.g. regression coefficient) AND variation (e.g. standard deviation) or associated estimates of uncertainty (e.g. confidence intervals)
☐	☒	For null hypothesis testing, the test statistic (e.g. *F*, *t*, *r*) with confidence intervals, effect sizes, degrees of freedom and *P* value noted<br>*Give P values as exact values whenever suitable.*
☒	☐	For Bayesian analysis, information on the choice of priors and Markov chain Monte Carlo settings
☒	☐	For hierarchical and complex designs, identification of the appropriate level for tests and full reporting of outcomes
☒	☐	Estimates of effect sizes (e.g. Cohen's *d*, Pearson's *r*), indicating how they were calculated

Our web collection on statistics for biologists contains articles on many of the points above.

Software and code

Policy information about availability of computer code

Data collection	The following softwares were used for data collection: flow cytometry (MacsQuant (10 and VYB, Miltenyi, Bergisch Gladbach Germany) and MacsQant software for FACS analysis, Zeiss ZEN microscope software for TPLSM image acquisition, Tecan microplate reader for permeability assay and ELISA.
Data analysis	The acquired images were processed using ImageJ software to enhance the contrast.<br>FACS data were analyzed with MacsQant software.<br>All statistical analyses were conducted with Prism 8.0c version.<br>Quantification of the total volume of neutrophils inside the sinus area over time was done using Imaris software {v9.5.1, Bitplane AG}.

For manuscripts utilizing custom algorithms or software that are central to the research but not yet described in published literature, software must be made available to editors and reviewers. We strongly encourage code deposition in a community repository (e.g. GitHub). See the Nature Research guidelines for submitting code & software for further information.

Data

Policy information about availability of data

All manuscripts must include a data availability statement. This statement should provide the following information, where applicable:

- Accession codes, unique identifiers, or web links for publicly available datasets
- A list of figures that have associated raw data
- A description of any restrictions on data availability

Data have been deposited in the GenBank (Gene Expression Omnibus; GEO) with the accession code GSE143978 {https://www.ncbi.nlm.nih.gov/geo/query/acc.cgi?acc=GSE143978}.
All relevant data needed to evaluate the conclusions are available within the article and Supplementary Information. All other relevant data are provided in the

source data file, or available from the corresponding author on request.

Field-specific reporting

Please select the one below that is the best fit for your research. If you are not sure, read the appropriate sections before making your selection.

☒ Life sciences ☐ Behavioural & social sciences ☐ Ecological, evolutionary & environmental sciences

For a reference copy of the document with all sections, see nature.com/documents/nr-reporting-summary-flat.pdf

Life sciences study design

All studies must disclose on these points even when the disclosure is negative.

Sample size	No sample-size calculations were performed. Sample size was determined to be adequate based on the magnitude and consistency of measurable differences between groups. To avoid unethical use of mice, we determined the number of animal use according to field standards and reproducibility achieved among different mice.
Data exclusions	Mice showing exceptional values in all measured parameters (most probably due to technical reasons or due to poor health status) were excluded from the study. Along the entire study, 12 mice of >1000 were thus excluded.
Replication	Replicate experiments were successful. Each experiment was controlled by technical and biological replicates as indicated in figure legends. Data was summarized from at least 2 independent experiments with minimum of 3 mice per experimental group.
Randomization	Mice were assigned according to their genotype. Litter mates and sex-matched animals were used whenever possible. All other parameters are random.
Blinding	The investigators were not blinded to mouse genotypes or sample type during experiments. Data reported are based on quantitative flow cytometry and image quantification analysis. Blinding is not relevant to this study since mice usage is based on their genotypes and therefore cannot be blind.

Reporting for specific materials, systems and methods

We require information from authors about some types of materials, experimental systems and methods used in many studies. Here, indicate whether each material, system or method listed is relevant to your study. If you are not sure if a list item applies to your research, read the appropriate section before selecting a response.

Materials & experimental systems

n/a	Involved in the study	
☐	☒	Antibodies
☒	☐	Eukaryotic cell lines
☒	☐	Palaeontology and archaeology
☐	☒	Animals and other organisms
☒	☐	Human research participants
☒	☐	Clinical data
☒	☐	Dual use research of concern

Methods

n/a	Involved in the study	
☒	☐	ChIP-seq
☐	☒	Flow cytometry
☒	☐	MRI-based neuroimaging

Antibodies

Antibodies used	Antibodies used in our study: (Ab name, clone, Supplier name (Origin), Catalog number, lot number)<br>For neutrophil staining, we used anti-Ly6G-APC (Clone: A18, Origin: Biogems, Catalog number: 83112-80-100, lot number: 80G221683112) and anti-CD11b-FITC (M1/70, Biolegend, 101206, B246148). For monocyte staining, we used anti-Ly6C-PE-Cy7 (HK1.4,BioLegend, 128018, B242951) and anti-CD11b-FITC (M1/70, BioLegend). For detection of depleted neutrophils, we used GR-1-APC (RB6-8C5, BioLegend). For sorting neutrophils, we used a combination of CD45-PE (30-F11, 103106, B196478)/Ly6G-APC/CD11b-FITC (all from BioLegend). For lymphocytes staining, we used anti-CD4-FITC(GK15, 103106, B266671) and anti-B220-PE(RA3-6B2, 103208, B250169) all from BioLegend).<br>For MCT4 expression, we stained with rabbit anti mouse MCT4 (H-90, Santa Cruz, sc-50329, K0315) followed by anti-rabbit PE (Jackson ImmunoResearch, 711-116-152, 140688). For MCT1 expression, we stained with rabbit anti-mouse MCT1 (M-45, Santa Cruz, sc-50325, B2316) followed by anti-rabbit PE (catalog number: 711-116-152, lot number: 140688; Jackson ImmunoResearch). HIF-1-alpha was identified using conjugated anti-h/m HIF-1 alpha-PE (241812, R&D, IC1935P, AALY0416121). For LDHA expression, we stained with rabbit anti-mouse LDHA (EPR1564, Abcam, ab101562, GR176934-3) followed by anti-rabbit PE (Jackson ImmunoResearch).<br>Sinusoidal endothelial cells were identified by staining with anti-CD31-PE-Cy7(390, 102418, B212262), anti-Sca-1-PE (D7,108108, B188263) or Pacific Blue (D7, 108120, B211125) and anti-CD45-APC (30-F11, 103112, B210891) (all from Biolegend). For GPR81

	expression, we stained with rabbit anti-mouse GPR81 (Novus, NLS2095, 41149) followed by anti-rabbit PE (Jackson ImmunoResearch). For VE-Cadherin expression, we stained with anti-VE-Cadherin-BV421 conjugated antibody (11D4.1, BD Bioscience, 562795, 4318552).
Validation	Antibodies specificity was validated according to the manufacturer's instructions and all antibodies used are common and are largely described in the literature. To ensure specific binding, a known negative population was used for validation as a population that does not bind the antibody.<br>The Fluorescence Minus One Control (FMO) used to identify and gate cells in the context of data spread due to the multiple fluorochromes in a given panel. In other words, staining without addition the"target" Ab (like MCT1, MCT4, LDHA, etc..) was considered to be the baseline for gating the positive population.

Animals and other organisms

Policy information about studies involving animals; ARRIVE guidelines recommended for reporting animal research

Laboratory animals	C57BL/6 mice were purchased from Harlan Laboratories (Rehovot, Israel). HIF-1 alpha mice (B6.129- Hif1atm3Rsjo/J), LysM-Cre mice (B6.129P2-Lyz2tm1(cre)Ifo/J), gp91phox-/- (B6.129S-Cybbtm1Din/J) mice and transgenic Ly6a(Sca-1)-eGFP mice were purchased from Jackson Laboratories.<br>Transgenic LysM-GFP mice were kindly provided by G. Shachar (Weizmann Institute, Israel). GPR81-/- and GPR81-RFP mice were kindly provided by S. Offermanns (Department of Pharmacology, Max-Planck-Institute for Heart and Lung Research, Germany). Catchup (Ly6G tdTomato) mice were kindly provided by M. Gunzer (University Duisburg-Essen, Germany). Conditional mutants carrying loxP-flanked GPR81 were kindly provided by Z. Gerhart-Hines (University of Copenhagen, Denmark).<br>All mutated or transgenic mouse strains were compared to strain matched wild-type C57BL/6 sex-and age-matched controls.<br>All wild-type, mutated or transgenic mouse strains were 8-10-weeks-old mice, both male and female, were used for all experiments.<br>Mice were kept in a specific pathogen-free, temperature-controlled (22°C ± 1°C) facility on a standard 12-h light/dark cycle at the Weizmann Institute of Science.
Wild animals	Not used in this study.
Field-collected samples	This study did not involve sample collection from the field.
Ethics oversight	Breeding and all experimental procedures were monitored by the Veterinary Resources Unit of the Weizmann Institute and were approved by the Institute Animal Care and Use Committee (IACUC).

Note that full information on the approval of the study protocol must also be provided in the manuscript.

Flow Cytometry

Plots

Confirm that:

- [x] The axis labels state the marker and fluorochrome used (e.g. CD4-FITC).
- [x] The axis scales are clearly visible. Include numbers along axes only for bottom left plot of group (a 'group' is an analysis of identical markers).
- [x] All plots are contour plots with outliers or pseudocolor plots.
- [x] A numerical value for number of cells or percentage (with statistics) is provided.

Methodology

Sample preparation	Samples were prepared as described in the methods section. Briefly, mice were sacrificed and bone marrow and liver were removed. Peripheral blood was collected from the heart using heparinized syringes.<br>Bone marrow were obtained by flushing long bones with PBS and filtered before staining.<br>Isolated peripheral blood and liver cells underwent red blood cell lysis (R7577, Sigma) before staining.<br>For bone marrow endothelial cell staining, bone marrow cells were flushed with Liver digest medium (LDM; Invitrogen) supplemented with 0.01% DNase 1 (Roche) and mechanically crushed by mortar and pestle in the same medium, followed by 30 min digestion at 37 °C with shaking. Following incubation time, cells were washed twice with PBS. Next, cells underwent red blood cell lysis, filtered and washed extensively before staining.
Instrument	MacsQuant (10 and VYB, Miltenyi, Bergisch Gladbach Germany)
Software	Data were analyzed with MacsQant software and for statistical analysis Prism 8.0c, R were used.
Cell population abundance	Bone marrow neutrophils sorting was applied in this study.<br>BM single cell suspensions were sorted gating on CD45+/CD11b+/Ly6Ghigh cells using SORP-FACSAriaII machine with a 100 µm nozzle. Dead cells were excluded on the basis of DAPI incorporation reaching purities greater than 94%.<br><br>Bone Marrow neutrophil isolation for in vitro experiments was applied in this study. Flushed BM cells (from WT and mutated mice) were resuspended in 200 µl MACS buffer (2 mM EDTA, 0.5 % BSA in PBS) and BM neutrophils were isolated according to the protocol of 'Neutrophil isolation kit, mouse' by Miltenyi. The yields were approximately 8 miilions cells in total (for 6

bones of two legs), and the purity was > 94%.

Gating strategy

Specific gating strategies are specified in the figure legends and/or methods section. Briefly, Double positive gate of FSC-H and SSC-H, based on this properties gating for leukocytes was performed.
Next, markers for specific cell populations were used to define populations. In most experiments, murine neutrophils were investigated, which were defined as CD11b high and Ly6G high. Murine monocytes were defined as CD11b intermediate and Ly6C intermediate. Murine bone marrow sinusoidal endothelial cells were defined as CD45 negative, CD31 positive and Sca-1 negative. Murine bone marrow arterials endothelial cells were defined as CD45 negative, CD31 positive and Sca-1 positive.

For FACS staining, we used the fluorescence minus one (FMO) as a negative control, which contains all the fluorochromes except for the one that is being measured. The FMO in the manuscript is presented as “secondary only”.

☒ Tick this box to confirm that a figure exemplifying the gating strategy is provided in the Supplementary Information.
